# Quantitative characterization of protein–protein complexes involved in base excision DNA repair

**DOI:** 10.1093/nar/gkv569

**Published:** 2015-05-26

**Authors:** Nina A. Moor, Inna A. Vasil'eva, Rashid O. Anarbaev, Alfred A. Antson, Olga I. Lavrik

**Affiliations:** 1 Institute of Chemical Biology and Fundamental Medicine, Siberian Branch of the Russian Academy of Sciences, Novosibirsk 630090, Russia; 2 Structural Biology Laboratory, Department of Chemistry, University of York, York YO10 5DD, UK; 3 Department of Natural Sciences, Novosibirsk State University, Novosibirsk 630090, Russia

## Abstract

Base Excision Repair (BER) efficiently corrects the most common types of DNA damage in mammalian cells. Step-by-step coordination of BER is facilitated by multiple interactions between enzymes and accessory proteins involved. Here we characterize quantitatively a number of complexes formed by DNA polymerase β (Polβ), apurinic/apyrimidinic endonuclease 1 (APE1), poly(ADP-ribose) polymerase 1 (PARP1), X-ray repair cross-complementing protein 1 (XRCC1) and tyrosyl-DNA phosphodiesterase 1 (TDP1), using fluorescence- and light scattering-based techniques. Direct physical interactions between the APE1-Polβ, APE1-TDP1, APE1-PARP1 and Polβ-TDP1 pairs have been detected and characterized for the first time. The combined results provide strong evidence that the most stable complex is formed between XRCC1 and Polβ. Model DNA intermediates of BER are shown to induce significant rearrangement of the Polβ complexes with XRCC1 and PARP1, while having no detectable influence on the protein–protein binding affinities. The strength of APE1 interaction with Polβ, XRCC1 and PARP1 is revealed to be modulated by BER intermediates to different extents, depending on the type of DNA damage. The affinity of APE1 for Polβ is higher in the complex with abasic site-containing DNA than after the APE1-catalyzed incision. Our findings advance understanding of the molecular mechanisms underlying coordination and regulation of the BER process.

## INTRODUCTION

Cellular genomic DNA permanently suffers various molecular lesions, due to endogenous and exogenous factors (1). In order to maintain genome integrity, cells have evolved DNA repair systems to efficiently correct DNA damage. Several specific pathways of DNA repair in mammalian cells have been established (1,2). The primary means for correcting the most common types of damage such as apurinic/apyrimidinic (AP) sites (created through N-glycosidic bond cleavage), modified bases and single strand breaks (SSBs) is base excision repair (BER). The BER process can proceed along one of two sub-pathways that are differentiated by the size of the repair patch and by enzymes and accessory proteins/cofactors involved. These two sub-pathways are termed ‘short-patch BER’ (SP BER) and ‘long-patch BER’ (LP BER) (1,2). The core steps of BER are catalyzed by a number of enzymes (1,2). DNA glycosylases recognize and remove altered bases, creating an intact or cleaved AP site. The intact AP site is incised by the endonucleolytic activity of the AP endonuclease 1 (APE1). Terminal blocking groups of the DNA strand break intermediates generated by either bifunctional DNA glycosylases or APE1, or direct action of reactive oxygen species and physical agents are removed by DNA polymerase β (Polβ; via its 5′-deoxyribose phosphate lyase activity), APE1 (via its 3′-phosphodiesterase and 3′-phosphatase activities), polynucleotide kinase 3′-phosphatase (PNKP) and aprataxin. The end-processing step is followed by gap filling catalyzed by Polβ during the SP BER. During the LP BER, DNA synthesis is mediated by Polβ, Polδ and/or Polϵ, and the displaced DNA strand is cleaved by flap endonuclease 1 (FEN1). At the final step, the integrity of the DNA backbone is completed by DNA ligases: LigI in LP BER and LigIIIα in SP BER.

According to the ‘passing-the-baton’ (or ‘substrate channeling’) model of the BER process, DNA intermediates in each of the two BER sub-pathways are processed and then passed from one enzyme to another in a coordinated fashion (1,2). Step-by-step coordination of BER is facilitated by multiple protein–protein interactions (1–3) that involve BER enzymes and accessory proteins such as XRCC1, PARP1, PARP2, TDP1, PCNA and aprataxin (Supplementary Table S1). XRCC1 (X-ray repair cross-complimenting protein 1) functions as a non-enzymatic, scaffold protein that directly interacts with, stabilizes, and stimulates multiple enzymatic components of the BER process by using its N-terminal domain and two BRCT (BRCA1 C-terminal) domains (3–5). PARP1 is an abundant nuclear protein activated by binding to SSBs generated directly by irradiation or indirectly during BER (3,6). PARP1 modifies itself and other target proteins with branched chains of poly(ADP-ribose), contributing to regulation of many cellular processes (3,6). PARP1 and its closest homolog PARP2 interact physically with BER proteins and DNA intermediates, and modulate functions of the BER enzymes APE1, Polβ and FEN1, thus regulating the efficiency of BER (7–11). TDP1 functions as a general 3′-DNA phosphodiesterase catalyzing hydrolysis of 3′-phosphotyrosyl bonds, 3′-abasic sites, 3′-phosphoglycolates and 3′-dRP lesions (12). Recently TDP1 was shown to initiate APE1-independent repair of AP sites in different DNA structures, thus expanding the ability of the BER process (13). Protein–protein interactions between BER enzymes and other protein factors have been mostly characterized by biochemical and immunological approaches. These qualitative studies have led to the identification of a number of interacting partners and specific peptide sequences that are essential for protein recognition (Supplementary Table S1). It remains to be seen whether BER proteins are organized into constitutive complexes or whether they form sequential transient assemblies (3), justifying quantitative thermodynamic and kinetic studies that would help understanding the molecular mechanisms of BER coordination.

In the present study, interactions between BER enzymes (APE1 and Polβ) and accessory proteins (XRCC1, PARP1 and TDP1) were examined and characterized quantitatively by several biophysical techniques. Using fluorescence-based approaches, the relative binding affinities of APE1, Polβ, PARP1 and XRCC1 for various protein partners were determined and compared for the first time in the absence and presence of model DNA intermediates of BER. Fluorescence resonance energy transfer (FRET) experiments enabled to confirm physical interactions between the protein pairs and to follow DNA-induced rearrangements of the protein–protein complexes. The oligomeric state of certain individual proteins and complexes and stability of the complexes were additionally explored by light scattering techniques. Finally, functional implications of the protein–protein interactions examined are discussed in the context of regulation and coordination of BER.

## MATERIALS AND METHODS

### Protein expression and purification

Recombinant human apurinic/apyrimidinic endonuclease 1 (APE1), rat DNA polymerase β (Polβ) and human tyrosyl-DNA phosphodiesterase 1 (TDP1) were produced by expression in *Escherichia coli* BL21(DE3)pLysS and purified as described previously (14–16). Human protein XRCC1 and human poly(ADP-ribose) polymerase 1 (PARP1) were produced by expression in *E. coli* Rosetta(DE3) and purified as described (17,18). A 24-kDa fragment of human PARP1 (p24) was produced by expression in *E. coli* BL21(DE3)pLysS and purified following the procedure described for PARP1 (18). The APE1 and Polβ expression vectors were kindly provided by Prof. S.H. Wilson (National Institute of Health, North Carolina, USA). The TDP1 and XRCC1 expression vectors were generous gifts from Dr K.W. Caldecott (University of Sussex, UK) and Dr J.P. Radicella (UMR217 CNRS/CEA, France). The plasmid constructs used to express PARP1 and p24 were kindly provided by Dr M. Satoh (Laval University, Quebec City, Canada). The purified proteins were dialyzed against a solution containing 50 mM Tris-HCl, pH 8.0, 200 mM NaCl, 1 mM EDTA, 5 mM DTT and 40% glycerol, and stored at −30°C.

### Fluorescent labeling of APE1, Polβ, PARP1, XRCC1 and TDP1

The protein to be labeled was dialyzed against a solution containing 100 mM MES, pH 7.0, 150 mM NaCl and 2 mM DTT, using a Viva-spin microconcentrator, with five washing steps followed by a final step against the same solution containing no DTT. 5-(4,6-Dichlorotriazinyl)aminofluorescein (DTAF; Sigma-Aldrich), the N-succinimidyl ester of 5(6)-carboxytetramethylrhodamine (TMR-SE; Sigma-Aldrich) or the N-succinimidyl ester of 5(6)-carboxyfluorescein (FAM-SE; Sigma-Aldrich) was dissolved in dimethyl sulfoxide at 10 mM concentration and added to the protein solution. The reaction mixtures contained 100 mM MES, pH 7.0, 150 mM NaCl, 100 μM protein, and various concentrations of the labeling reagent ranging from 160 μM to 1 mM. The reaction was allowed to proceed for 2–17 h at 4°C in the dark. The reaction mixture was diluted by addition of four volumes of a solution containing 100 mM MES, pH 7.0, 150 mM NaCl and 10 mM DTT, and centrifuged at 10 000 rpm for 15 min. The supernatant was dialyzed exhaustively against a solution containing 100 mM HEPES, pH 8.0, 200 mM NaCl and 10 mM DTT, to remove the free dye. The labeled proteins were stored at −30°C in a solution containing 50 mM HEPES, pH 8.0, 100 mM NaCl, 5 mM DTT and 40% glycerol. The extent of protein labeling was quantified by determining dye and protein amounts in the sample. The dye concentration was measured spectrophotometrically using the absorption coefficients of 68×10^3^ M^−1^cm^−1^ at 494 nm for 5(6)-FAM and 65×10^3^ M^−1^cm^−1^ at 555 nm for 5(6)-TAMRA (TMR) (19). The protein concentration was determined using the Bradford assay (20). The enzymatic activities of Polβ in DNA synthesis and of APE1 in endonucleolytic DNA cleavage were verified as detailed in Supplementary Data.

### Fluorescence studies of protein–protein interactions

Binding of BER proteins to each other was examined by fluorescence titration experiments. Fluorescence intensities of solutions of the FAM(TAF)-labeled protein (at a fixed concentration) in binding buffer were measured in the absence and presence of various concentrations of the potential interaction partner. The binding buffer contained 50 mM HEPES, pH 8.0, 100 mM NaCl and 4 mM DTT. Samples (200 μl) were measured in Corning™ black 96-well polypropylene assay plates. All the measurements were carried out in duplicates for each specific condition, and the average values of fluorescence were taken. Fluorescence intensity measurements and data analysis were performed using a POLARstar OPTIMA multifunctional microplate reader and MARS Data Analysis Software (BMG LABTECH GmbH, Germany). The fluorescent probes were excited at 485 nm (485BP1 filter), and the fluorescence intensity was detected at the emission maximum (520 nm; EM520 filter). The data were plotted (F versus C) and fitted by four-parameter logistic equation:

\begin{equation*} {F} = {F}_0 + ({F}_\infty - {F}_0 )/[1 + ({\rm EC}_{50} /{C})^{n} ] \end{equation*}

where *F* is the measured fluorescence intensity of a solution containing the FAM(TAF)-labeled protein at a given concentration (*C*) of the binding partner, *F*_0_ is the fluorescence of a solution of the labeled protein alone, *F*_∞_ is the fluorescence of the labeled protein saturated with the partner, EC_50_ is the concentration of the binding partner at which *F* − *F*_0_ = (*F*_∞_ − *F*_0_)/2, and n is the Hill coefficient, which denotes the steepness (slope) of the nonlinear curve.

To detect protein–protein interactions by the FRET approach, the fluorescence intensity of the FAM-labeled protein (donor-labeled probe) was measured in the absence and presence of various concentrations of the TMR-labeled protein (acceptor probe). Measurements were performed in three titration series: (i) donor-labeled probe + unlabeled partner; (ii) unlabeled probe + acceptor-labeled partner; (iii) donor-labeled probe + acceptor-labeled partner (FRET pair). The raw *F*_a_ value measured in the series (ii) was subtracted from the raw *F*_da_* value in the series (iii) at each titration point to obtain the corrected value of the donor fluorescence in the presence of the acceptor (*F*_da_). FRET efficiency (*E*) was calculated from the fractional decrease of the donor fluorescence (*F*_d_) due to the presence of the acceptor (*F*_da_): *E* = 1 – *F*_da_/*F*_d_. The donor–acceptor distance *R* in the complex was calculated using the equation: *R* = *R*_0_(*E*^−1^ – 1)^1/6^ where *R*_0_ is the Förster radius of a given dye pair (distance at which energy transfer is 50%). For the FAM and TMR pair, *R*_0_ corresponds to 55 Å (21).

In studying the effects of model DNA substrates (prepared as described in Supplementary Data) on the protein–protein interactions, the FAM-labeled protein (40 nM) was premixed with the respective DNA, and the fluorescence intensity observed was taken as a starting *F*_0_ value. Conditions for the formation of each ternary complex were optimized by varying the concentration of the DNA in the range of 120 to 400 nM. Titration of the FAM-labeled APE1 in the presence of various DNAs was performed in binding buffer supplemented with 10 mM EDTA (to suppress the endonuclease and the 3′-exonuclease activities of APE1).

### Size-exclusion chromatography with multi-angle laser light scattering (SEC-MALLS)

SEC-MALLS experiments were performed on a Shimadzu HPLC system with the SPD20A UV/Vis detector linked to a Wyatt Dawn HELEOS-II 18-angle light-scattering detector and a Wyatt Optilab rEX refractive index monitor. SEC was carried out on a Phenomenex HPLC column BioSep-SEC-S 3000, equilibrated and run at a flow rate of 1 ml/min in a solution consisting of 25 mM HEPES, pH 7.5, 200 mM NaCl and 2 mM DTT. 100-μl samples of individual proteins (APE1, Polβ, and XRCC1) at concentrations ranging from 2.1 to 4.2 mg/ml or equimolar mixtures of two (or three) proteins were incubated at 4°C for 2 h and injected using an SIL-20A Autosampler. The SEC-MALLS experiments were performed at room temperature. The profile line was monitored using a UV monitor at 280 nm and a refractive index detector at 690 nm. The data were analyzed with the Astra V software (Wyatt Technology) using a refractive index increment (dn/dc) value of 0.190.

### Dynamic light scattering (DLS) experiments

DLS experiments were performed at 24 ± 0.1°C using a Malvern Instrument's Zetasizer Nano ZS. Samples of individual proteins (APE1, Polβ, and XRCC1) or equimolar mixtures of proteins at final concentrations of 6 μM were prepared in a solution consisting of 25 mM HEPES, pH 7.5, 100 mM NaCl and 1 mM DTT. The measurements were performed using a 15-μl quartz cuvette. Each measurement was performed by accumulation of 11 scans, each with a duration of 10 s. The DTSv612 software (Malvern Instruments Ltd.) was used to analyze the acquired correlogram (correlation function versus time) and to derive the translational diffusion coefficient (D). Assuming particle sphericity, the hydrodynamic radius (*R*_H_) of the diffusing particles was calculated using the Stokes–Einstein equation: *R*_H_ = *kT*/6πηD where *k* is Boltzmann's constant, *T* is the absolute temperature, and η is the viscosity of the continuous phase, taken here as the viscosity of water at 297°K.

## RESULTS

### Detection and quantification of protein–protein interactions by fluorescence titration experiments

To detect complexes formed between the BER proteins and to obtain quantitative data for their interaction by fluorescence-based approaches, fluorescein-labeled and TMR-labeled proteins (APE1, Polβ, PARP1, XRCC1 and TDP1) were prepared using succinimidyl esters of 5(6)-carboxyfluorescein and 5(6)-carboxytetramethylrhodamine (FAM-SE, TMR-SE), and 5-(4,6-dichlorotriazinyl)-aminofluorescein (DTAF). Succinimidyl esters and dichlorotriazine are amine-reactive probes with strong pH dependence of the labeling reaction (19). In buffers with a neutral pH primary labeling of the terminal amino group may be achieved (19). Synthesis and characterization of the fluorescent-labeled proteins are described in Supplementary Data. Fully active enzymes and proteins with the labeling stoichiometry not exceeding 1 mol of label per mol of protein (Supplementary Table S2) were used to perform steady-state fluorescence experiments.

**Figure 1.**
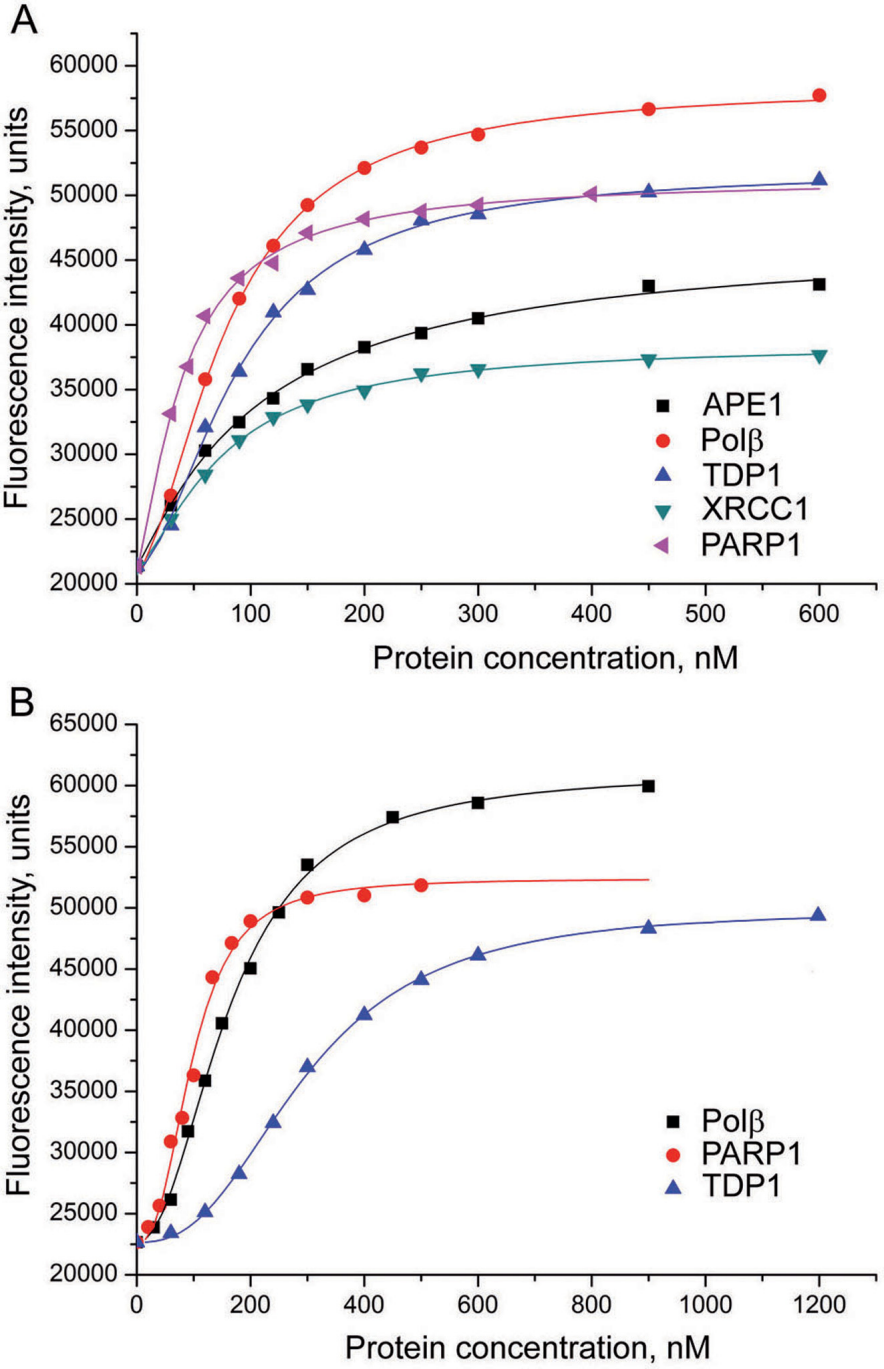
Fluorescence titration of FAM-APE1 (**A**) and FAM-Polβ (**B**) with various proteins. The FAM-labeled protein (40 nM) was excited at 485 nm in the absence or presence of increasing concentrations of the protein partner and the relative fluorescence intensities were monitored at 520 nm. Curves show the best fits of the four-parameter equation; *R*^2^ values meet or exceed 0.98. The data shown are representatives of at least three independent experiments.

Interaction between different enzymes and proteins involved in BER was studied by monitoring the change in the fluorescence intensity of a fluorescein-labeled protein (APE1, Polβ, XRCC1 or PARP1) in the presence of unlabeled proteins (potential binding partners) added at increasing concentrations. The titration curves obtained in experiments with FAM-labeled APE1 are shown in Figure 1A. The fluorescence intensity of FAM-APE1 increased in the presence of APE1, Polβ, XRCC1, PARP1 or TDP1, indicating that the local environment of the fluorophore changed upon protein–protein association. The fluorescence titration data show that APE1 is capable of forming both homo- and hetero-oligomeric complexes. Nonlinear regression analyses of the binding data (obtained by fitting to the four-parameter equation) yielded several parameters presented in Table 1 and Supplementary Table S3. The highest increase in fluorescence intensity at saturation (*F*_∞_/*F*_0_) was produced by Polβ binding (2.8-fold increase). Addition of XRCC1 had the weakest effect on FAM-APE1 fluorescence (1.9-fold increase at saturating concentrations). The half-maximal effective concentration of the protein partner (EC_50_) at which the fluorescence intensity is midway between the *F*_∞_ and *F*_0_ parameters, is an apparent equilibrium dissociation constant (24). The EC_50_ values determined for the FAM-APE1 complexes with APE1, Polβ, XRCC1, PARP1, and TDP1 varied from 45 to 120 nM (Table 1). They indicate a high affinity interaction of APE1 with PARP1 and the lowest affinity for APE1 self-association. The interaction of APE1 with Polβ and XRCC1 as well as its self-association were also detected by monitoring changes in fluorescence intensity of the TAF-labeled APE1 (data not shown). The EC_50_ values derived from the TAF-APE1 titration data were similar to the respective values determined for the FAM-APE1 complexes.

**Table 1.** Binding parameters of protein–protein interactions determined by fluorescence titration

Labeled protein^a^	Protein partner	EC_50_^b^, nM
FAM-APE1	APE1	120 ± 14
FAM-APE1	Polβ	84 ± 7
FAM-APE1	XRCC1	76 ± 9
FAM-APE1	PARP1	45 ± 5
FAM-APE1	p24	420 ± 55
FAM-APE1	TDP1	97 ± 8
FAM-Polβ	Polβ	160 ± 18
FAM-Polβ	PARP1	98 ± 8
FAM-Polβ	p24	670 ± 60
FAM-Polβ	TDP1	270 ± 36
FAM-PARP1	PARP1	130 ± 16
FAM-PARP1	p24	610 ± 40
FAM-PARP1	Polβ	110 ± 12
FAM-PARP1	XRCC1	100 ± 11
FAM-PARP1	TDP1	120 ± 15
TAF-XRCC1	XRCC1	73 ± 8
TAF-XRCC1	Polβ	25 ± 5
FAM-XRCC1 + gap-DNA	Polβ	23 ± 5
FAM-XRCC1	TDP1	80 ± 12

^a^Titration experiments were performed at a constant concentration of the fluorescein-labeled protein (40 nM); gap-DNA (a 32-mer 1-nt-gapped DNA shown in Supplementary Figure S1) was added at a 3-fold molar excess over the FAM-XRCC1.

^b^Parameters derived from the titration curves by fitting to the four-parameter equation, where EC_50_ is the half-maximal effective concentration of the protein partner, at which *F* − *F*_0_ = (*F*_∞_ − *F*_0_)/2. Values are the mean (± SD) of at least three independent experiments.

The titration curves obtained in experiments in which the fluorescence change of the FAM-labeled Polβ was monitored are presented in Figure 1B. Addition of unlabeled Polβ, PARP1 or TDP1 resulted in a significant increase in FAM-Polβ fluorescence (from 2.2- to 2.7-fold at saturating concentrations of various protein partners; Supplementary Table S3), indicating self-association of Polβ and its hetero-oligomerization with PARP1 and TDP1. The highest affinity was determined for the interaction of Polβ with PARP1 that was 2.7-fold higher than the affinity of Polβ for TDP1 (Table 1). Addition of APE1 had no effect on the FAM-Polβ fluorescence, in contrast to the significant change of the FAM-APE1 fluorescence induced by the interaction with Polβ. Titration of FAM-Polβ with XRCC1 revealed a slight increase in fluorescence intensity that was insufficient to allow us to determine the binding parameters (data not shown). Fluorescence of the TAF-labeled Polβ did not change in the presence of either APE1 or XRCC1, while a significant increase induced by Polβ binding was detected (data not shown).

**Figure 2.**
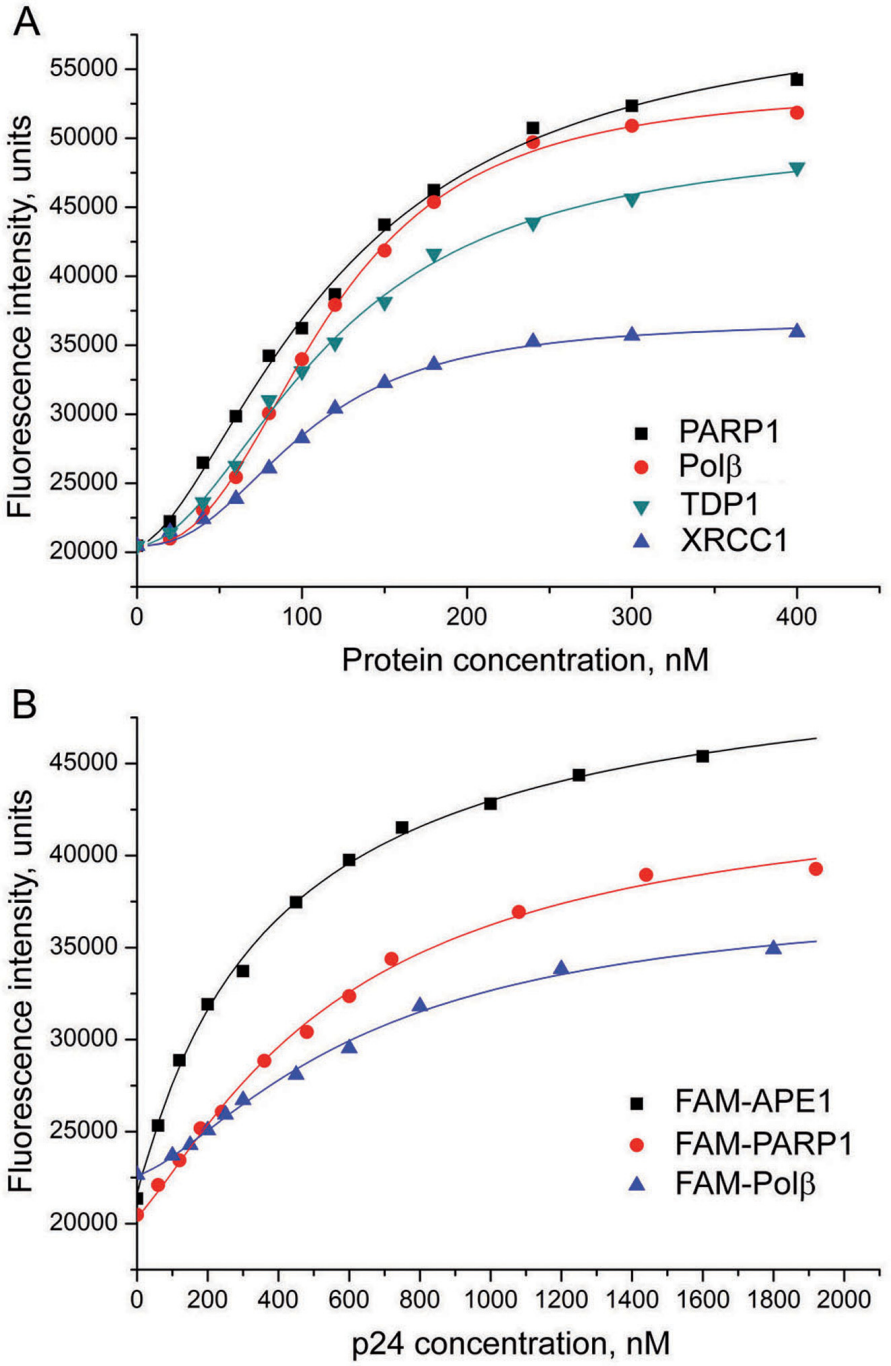
Fluorescence titration of FAM-PARP1 with PARP1, Polβ, XRCC1 and TDP1 (**A**), and of the FAM-labeled APE1, PARP1 and Polβ with p24 protein (**B**). The FAM-labeled protein (40 nM) was excited at 485 nm in the absence or presence of increasing concentrations of the binding partner and the relative fluorescence intensities were monitored at 520 nm. Curves show the best fits of the four-parameter equation (with *R*^2^ values exceeding 0.98).

The fluorescence intensity of FAM-PARP1 increased in the presence of four different proteins, namely PARP1, Polβ, XRCC1 or TDP1 (Figure 2A), while addition of APE1 had no effect (data not shown). Interestingly, the highest change of fluorescence intensity in titration experiments with the FAM-labeled PARP1 and the FAM-labeled Polβ was induced by self-association of the protein (Supplementary Table S3). The EC_50_ values determined for the four binding partners of FAM-PARP1 are comparable (Table 1), indicating that PARP1 can interact with homologous and many heterologous proteins with closely similar affinities. The apparent dissociation constants measured by titration of FAM-Polβ with PARP1 or of FAM-PARP1 with Polβ are practically identical, suggesting that the fluorescent labeling of either partner did not disturb their interaction.

The Hill coefficient (n) obtained from nonlinear regression analyses of the binding data was close to one when FAM-APE1 and FAM-PARP1 were titrated with the homologous protein partners (Supplementary Table S3), suggesting that the major homo-oligomeric form of APE1 and PARP1 is a dimer. The *n* values determined in the other experiments were significantly higher than one (in the range from 1.6 to 2.4), indicating the formation of multimeric complexes upon Polβ self-association and interaction of APE1, Polβ and PARP1 with the heterologous protein partners. All the proteins can homo-oligomerize, and their interaction could result in the formation of multimeric complexes of various stoichiometric combinations of the components. Reaction schemes for such sophisticated binding patterns are much more complicated than the reaction scheme described by the Hill equation (applied as the four-parameter function) that however is useful to quantify macromolecular interactions (22,23).

To compare binding affinities of different proteins for the full-length PARP1 and its N-terminal DNA binding fragment, the FAM-labeled APE1, Polβ and PARP1 were titrated with the p24 protein. This apoptotic fragment of PARP1 (residues 1–214) is composed of two zinc-finger subdomains of the DNA binding domain. The titration curves presented in Figure 2B provide evidence for p24 binding to all three proteins. The EC_50_ values derived from the titration data are presented in Table 1. Their analysis shows that affinity of p24 for the different proteins is significantly (5–9-fold) lower than that of PARP1.

**Figure 3.**
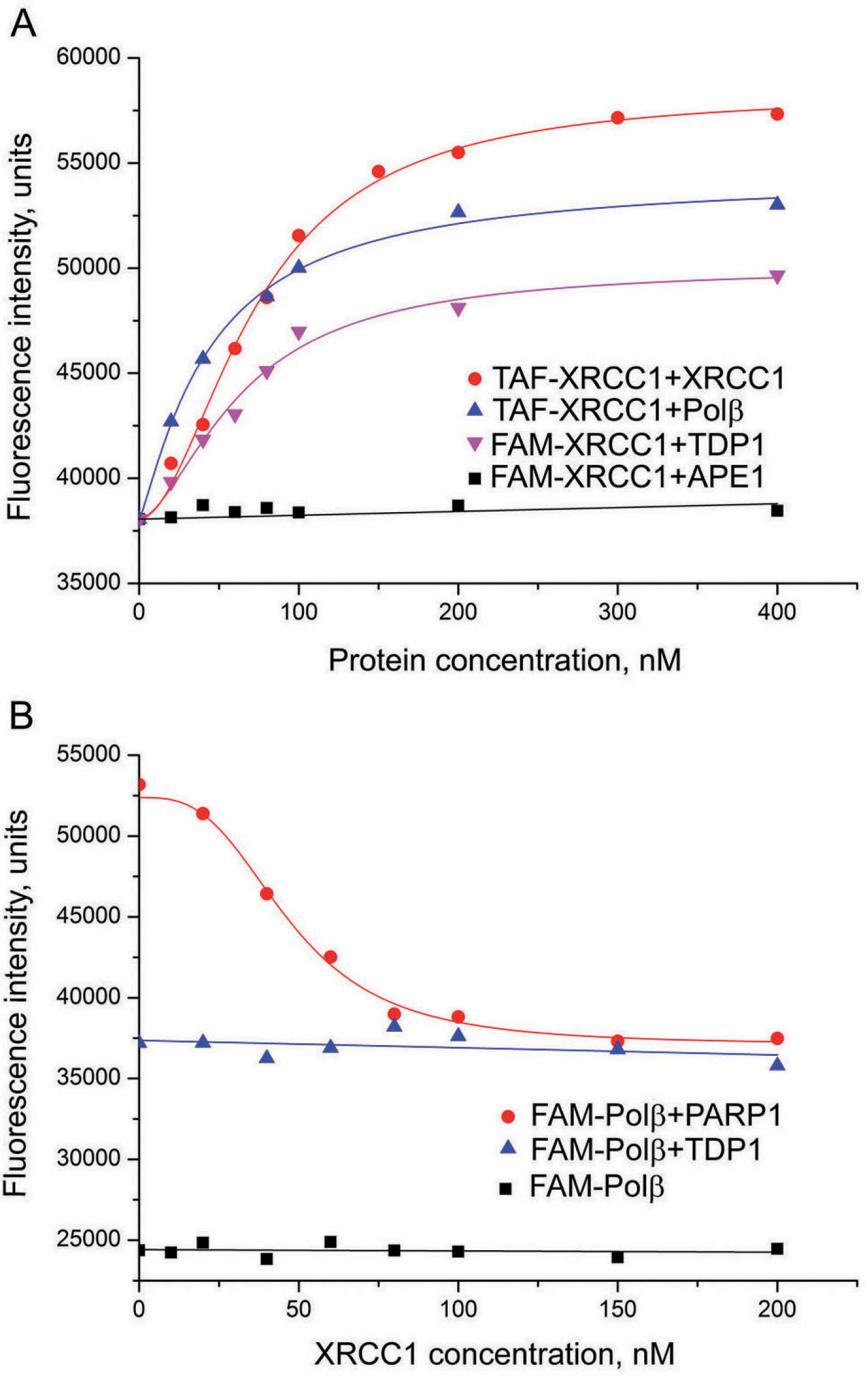
Detection of XRCC1 complexes (binary and ternary) by fluorescence titration of FAM(TAF)-labeled XRCC1 with XRCC1, APE1, Polβ or TDP1 (**A**) and of FAM-Polβ with XRCC1 in the absence and presence of PARP1 or TDP1 (**B**). The labeled protein (40 nM FAM-XRCC1, TAF-XRCC1 or FAM-Polβ, separately or mixed with 80 nM PARP1/TDP1) was excited at 485 nm in the absence or presence of increasing concentrations of the protein partner and the relative fluorescence intensities were monitored at 520 nm. Curves show the best fits of the four-parameter equation (with *R*^2^ values exceeding 0.97).

Titration experiments with the FAM- and TAF-labeled XRCC1 enabled us to detect self-association of XRCC1 and its interaction with Polβ and TDP1 (Figure 3A). Addition of APE1 had no effect on the fluorescence intensity of either FAM-XRCC1 or TAF-XRCC1. Polβ binding induced an increase in the fluorescence intensity of FAM-XRCC1 only in the presence of a BER intermediate, a 1-nt-gapped DNA (gap-DNA), suggesting conformational modulation of the XRCC1-Polβ interaction by the DNA substrate of Polβ. The EC_50_ values determined for various XRCC1 complexes indicate considerably tighter binding of XRCC1 to Polβ than to itself, or to APE1, PARP1 or TDP1 (Table 1). It should be noted, that the EC_50_ value determined for the XRCC1 complex with Polβ is 1.7-fold below the concentration of the labeled molecule and therefore represents the upper limit of the apparent equilibrium dissociation constant.

Attempts were made to detect the formation of ternary protein–protein complexes by fluorescence titration experiments. The FAM-labeled Polβ was titrated with XRCC1 in the absence and presence of PARP1 or TDP1 (Figure 3B). The initial fluorescence intensity of FAM-Polβ was significantly higher in the presence of PARP1 or TDP1 than in their absence due to the formation of the respective binary complexes. Addition of XRCC1 to FAM-Polβ alone produced no detectable signal, while it decreased the fluorescence intensity of the FAM-Polβ complex with PARP1 to a new stable level (at sub-saturating concentrations of XRCC1) that was midway between the initial intensities of the complex and FAM-Polβ. The effect observed can be explained by the formation of an equilibrium mixture of the binary FAM-Polβ·XRCC1 and FAM-Polβ·PARP1 complexes, since full displacement of PARP1 from the preformed FAM-Polβ·PARP1 complex by XRCC1 should decrease the fluorescence intensity to the level of the FAM-Polβ·XRCC1 complex (i.e. to the initial intensity of FAM-Polβ). Formation of the ternary FAM-Polβ·PARP1·XRCC1 complex was not clear, but could not be excluded. The fluorescence intensity of the FAM-Polβ complex with TDP1 was practically constant upon addition of increasing XRCC1 concentrations, suggesting no competition between XRCC1 and TDP1 for binding to FAM-Polβ despite the significantly (10-fold) higher affinity of XRCC1 than of TDP1 for Polβ. The data unambiguously indicate the formation of the ternary FAM-Polβ·TDP1·XRCC1 complex.

### Characterization of protein–protein complexes by steady-state FRET binding assays

FRET refers to a quantum effect between a given pair of fluorophores, i.e. a fluorescent donor and an acceptor, where, upon excitation of the donor, energy is transferred from the donor to the acceptor via dipole–dipole coupling (24). FRET is characterized by the efficiency of the energy transfer (E), which is a function of the inverse sixth power of the distance (R) between the two fluorophores [*E* = *R*_0_^6^/(*R*_0_^6^ + *R*^6^)]. The distance at which energy transfer is 50% is known as the Förster distance (*R*_0_). *R*_0_ depends on the extent of spectral overlap between donor emission and acceptor absorption and is a unique property of a given FRET pair. The third key factor controlling the efficiency of energy transfer (along with the distance and the spectral overlap) is the relative orientation for dipole–dipole interaction (25). Although the contribution of the dipole orientation compromises FRET as an accurate measure of molecular distance, FRET is ideal to detect changes in binary macromolecular complexes induced by other interacting molecules (24). We have chosen fluorescein and tetramethylrhodamine as the most commonly used fluorescent donor-acceptor pair (19) to further confirm direct physical interactions between the BER proteins and to probe them for the formation of ternary complexes.

**Figure 4.**
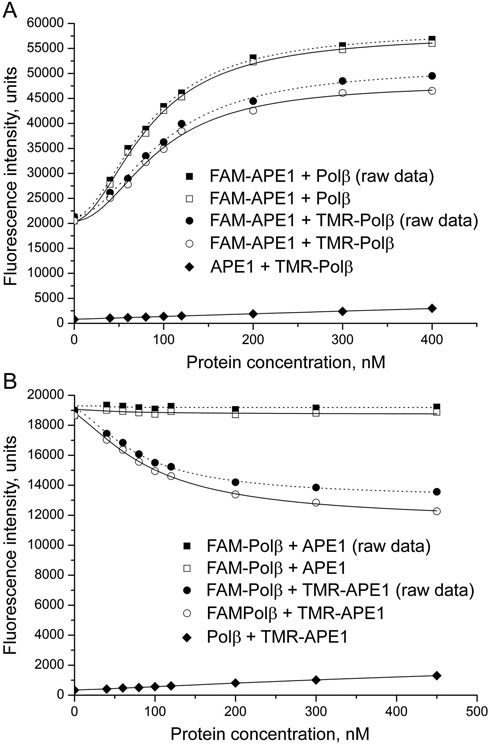
Detection of APE1-Polβ interaction by FRET. The FAM-labeled protein (40 nM FAM-APE1, panel **A**; 40 nM FAM-Polβ, panel **B**) was excited at 485 nm in the absence or presence of increasing concentrations of unlabeled or TMR-labeled partner (Polβ, panel A; APE1, panel B) and the relative fluorescence intensities were monitored at 520 nm. The efficiency of FRET was determined from the corrected data (open symbols) obtained from the raw data (filled circles and squares) by subtraction of the background or TMR fluorescence intensities that were measured in control series by titration of the unlabeled protein with the TMR-labeled partner (filled diamonds).

FAM-labeled APE1 was titrated with unlabeled Polβ or TMR-labeled Polβ (Figure 4A). The fluorescence was excited and monitored at the excitation and emission maxima of fluorescein. To correct for the fluorescence signal produced by the acceptor (especially in conditions where the acceptor was at significantly higher concentrations than the donor), unlabeled APE1 was titrated with TMR-Polβ in the control experiment. All three sets of experiments were performed in parallel in identical conditions. The fluorescence intensity of FAM-APE1 measured in the presence of TMR-Polβ was corrected by subtraction of the fluorescence intensity of TMR-Polβ (measured at the respective concentration in the presence of unlabeled APE1), and the corrected data were plotted to obtain the corrected binding curve (fitted to the four-parameter equation). The fluorescence intensity of FAM-APE1 increased less in the presence of TMR-Polβ than in the presence of Polβ, indicating that the donor-labeled and acceptor-labeled proteins participate in FRET. The efficiency of FRET was calculated from the fractional decrease of the fluorescence intensity of the donor due to the presence of the acceptor: *E* = 1 – *F*_da_/*F*_d_, where *F*_da_ and *F*_d_ are the FAM-APE1 fluorescence intensities measured in the presence of identical concentrations of TMR-Polβ (the corrected values) or Polβ respectively. The *F*_da_/*F*_d_ values determined at both low and sub-saturating concentrations of the protein partner were practically equal. This is obviously due to closely similar binding affinities of the unlabeled and TMR-labeled Polβ for FAM-APE1, as shown by comparing the respective EC_50_ parameters (Tables 1 and 2). The APE1-Polβ interaction was also detected with FRET when the inverse pair of donor- and acceptor-labeled proteins was used. The fluorescence intensity of FAM-Polβ did not change upon addition of APE1, while it decreased in the presence of TMR-APE1 (Figure 4B). The efficiency of FRET was considerably (2.3-fold) higher for the FAM-Polβ-TMR-APE1 pair than for the FAM-APE1-TMR-Polβ pair (Table 2), indicating that the relative orientation of two fluorophore probes in the complex of FAM-Polβ with TMR-APE1 was more favorable for FRET.

**Table 2.** Parameters of protein–protein interactions determined by FRET

Donor-labeled protein^a^	Acceptor-labeled protein	EC_50_^b^, nM	E^c^
FAM-APE1	TMR-Polβ	93 ± 8	0.16 ± 0.02
FAM-APE1 + XRCC1	TMR-Polβ	n.d.^d^	0.08 ± 0.02
FAM-APE1 + PARP1	TMR-Polβ	n.d.^d^	0.06 ± 0.02
FAM-Polβ	TMR-APE1	90 ± 10	0.37 ± 0.02
FAM-APE1	TMR-PARP1	51 ± 5	0.04 ± 0.02
FAM-PARP1	TMR-APE1	48 ± 6	0.28 ± 0.03
FAM-APE1	TMR-XRCC1	n.d.^d^	0.14 ± 0.02
FAM-Polβ	TMR-PARP1	110 ± 13	0.22 ± 0.04
FAM-PARP1	TMR-Polβ	110 ± 11	0.28 ± 0.02
FAM-Polβ	TMR-XRCC1	32 ± 4	0.10 ± 0.02
FAM-XRCC1	TMR-Polβ	31 ± 5	0.41 ± 0.02
FAM-PARP1	TMR-XRCC1	110 ± 11	0.22 ± 0.03
FAM-Polβ	TMR-TDP1	n.d.^d^	0.10 ± 0.02
FAM-XRCC1	TMR-TDP1	n.d.^d^	0.03 ± 0.02
FAM-APE1	TMR-APE1	130 ± 10	0.05 ± 0.03
FAM-Polβ	TMR-Polβ	170 ± 15	0.35 ± 0.02
FAM-PARP1	TMR-PARP1	130 ± 14	0.15 ± 0.03

^a^Titration experiments were performed at a constant concentration of the fluorescein-labeled protein (40 nM); XRCC1 or PARP1 was added (when indicated) at a 3-fold molar excess over the FAM-labeled protein.

^b^EC_50_ value for the TMR-labeled protein partner.

^c^Efficiency of FRET calculated from the experimentally determined fractional decrease of the fluorescence intensity: E = 1 – *F*_da_/*F*_d_, where *F*_da_ and *F*_d_ are fluorescence intensities of the donor-labeled protein measured in the presence of the acceptor-labeled or the unlabeled protein partner respectively. Relative intensity of fluorescence excited at 485 nm was monitored at 520 nm. F_da_ values were corrected for the contribution of the TMR-labeled protein (measured by titration of the unlabeled protein with the TMR-labeled partner). Values are the mean (± SD) of three independent experiments.

^d^The experiments were performed at sub-saturating concentrations of the TMR-labeled protein, and the EC_50_ value was not determined.

Analogous FRET measurements were performed for several other protein–protein complexes. Locations of the donor and acceptor fluorophores on the interacting proteins were interchanged, thus placing each dye in two different chemical environments. Binding parameters (EC_50_ values) and efficiencies of FRET as determined from these experiments are included in Table 2. Direct physical interaction of APE1 with PARP1 and of Polβ with XRCC1 was demonstrated by FRET when FAM-labeled PARP1 and XRCC1 were titrated with the respective TMR-labeled partners, while for the inverse donor-acceptor pairs FAM-APE1-TMR-PARP1 and FAM-Polβ-TMR-XRCC1 the efficiencies of FRET were significantly lower. On the contrary, closely similar FRET efficiencies were observed for the FAM-Polβ·TMR-PARP1 and FAM-PARP1·TMR-Polβ complexes. FRET signals were detected for the FAM-APE1-TMR-XRCC1, FAM-PARP1-TMR-XRCC1 and FAM-Polβ-TMR-TDP1 pairs. FAM-XRCC1 and TMR-TDP1 as well as the inverse donor-acceptor pair (data not shown) practically did not participate in FRET.

Homo-oligomeric protein complexes were also examined by FRET measurements. FRET signals were detected upon self-association of Polβ and PARP1 (Table 2). The efficiency of energy transfer between the FAM- and TMR-labeled Polβ was 2.3-fold higher than between the FAM- and TMR-labeled PARP1. This difference could be due to a significantly higher molecular size of PARP1 as compared to that of Polβ, thus reflecting the molecular distance between the interacting donor and acceptor fluorophores. No appreciable FRET was detected when the acceptor and donor probes were attached to APE1, whose size is comparable to that of Polβ. The data indicate that the efficiency of FRET is affected by the peculiar orientation of the donor and acceptor fluorophores rather than by their spatial separation.

To detect ternary protein–protein complexes, the efficiencies of FRET between FAM-APE1 and TMR-Polβ in the absence and presence of XRCC1 or PARP1 were compared (Table 2). Addition of both XRCC1 and PARP1 decreased the efficiency of FRET. The EC_50_ values were determined only in control experiments with unlabeled Polβ (data not shown), and a small increase in the binding affinity in the presence of both XRCC1 and PARP1 was detected. The results suggest the formation of the ternary complexes APE1·Polβ·XRCC1 and APE1·Polβ·PARP1, accompanied by structural rearrangements of the binary APE1·Polβ complex.

### Influence of BER intermediates on the protein–protein interactions

To explore possible modulation of protein–protein interactions by intermediates of DNA repair, fluorescence titration and FRET experiments were performed in the absence and presence of model DNA ligands (shown in Supplementary Figure S1). A double-stranded DNA with a synthetic abasic site (a tetrahydrofuran residue, THF) is an initial BER substrate of APE1 (AP-DNA). A 1-nucleotide (nt) gapped DNA with a 5′-THF group at the margin of the gap models a product of the APE1-catalyzed cleavage (incised AP-DNA) and a stable analog of Polβ substrate, which is not processed by the dRP lyase activity. A 1-nt-gapped DNA (gap-DNA) is a canonical substrate of the Polβ-catalyzed gap filling in SP BER, and a nicked DNA with an internal 5′-phosphate (nick-DNA) imitates a product of this reaction. Both incised AP-DNA and nick-DNA can serve as Polβ substrates in LP BER. APE1 and Polβ bind with highest affinity AP-DNA and gap-DNA respectively, and with relatively high affinity other DNAs tested (26). XRCC1 and PARP1 bind preferably 1-nt-gapped and nicked DNAs (27–30). Interaction of BER intermediates with FAM-labeled APE1, Polβ, XRCC1 and PARP1 was checked in independent experiments described in Supplementary Data (Supplementary Figure S2 and Supplementary Table S4). To insure saturation of each protein–protein complex with DNA, the concentration of DNA added first to the FAM-labeled protein (titrated further with a protein partner) was optimized in preliminary experiments. Results obtained at a 4-fold and a 10-fold molar excess of DNA over the FAM-protein were practically identical.

Effects produced by the model BER intermediates on the quantitative characteristics of protein–protein interactions (the apparent binding affinity constant and the FRET efficiency), were determined from these experiments (Table 3). The interaction between FAM-APE1 and Polβ was insignificantly affected by intact AP-DNA: the efficiency of FRET increased slightly (by 5%), while the binding affinity remained practically unchanged. The presence of incised AP-DNA resulted in the highest effect on the affinity (a 2-fold decrease), suggesting that APE1 and Polβ are more tightly bound to each other in the pre-incision than in the post-incision ternary complex. The gap-DNA-induced effect on the FAM-APE1 affinity for Polβ (a 1.3-fold decrease) was less pronounced as compared to that of nick-DNA (a 1.8-fold decrease), despite of a greater impact of gap-DNA on the efficiency of FRET. Parameters of FAM-APE1 binding to XRCC1 practically did not change in the presence of either intact or incised AP-DNA. However, the DNA-induced rearrangement of this complex was evident from the effects produced by gap-DNA: both the binding affinity and FRET efficiency increased appreciably (by 1.5-fold and 8% respectively). The binding affinity of FAM-APE1 for PARP1 decreased in the presence of each of the four DNAs tested, with the highest effects being produced by AP-DNA and nick-DNA. The efficiency of FRET in the FAM-APE1·TMR-PARP1 complex remained practically unchanged upon addition of gap-DNA and increased slightly in the presence of the other DNAs. Overall, these data indicate that the interaction of APE1 with Polβ, XRCC1 and PARP1 is modulated by BER intermediates, and the extent to which the strength of interaction is affected depends on the type of DNA damage to be processed.

**Table 3.** Effects of BER intermediates on the protein–protein interactions

FAM-labeled protein^a^	DNA^a^	Protein partner	Effect on binding affinity^b^	Effect on FRET efficiency^c^
FAM-APE1	AP-DNA	Polβ	1.1	+0.05
FAM-APE1	incised AP-DNA	Polβ	2.0	−0.06
FAM-APE1	gap-DNA	Polβ	1.3	−0.11
FAM-APE1	nick-DNA	Polβ	1.8	−0.07
FAM-APE1	AP-DNA	XRCC1	1.0	−0.01
FAM-APE1	incised AP-DNA	XRCC1	1.1	+0.02
FAM-APE1	gap-DNA	XRCC1	0.67	+0.08
FAM-APE1	AP-DNA	PARP1	1.7	+0.05
FAM-APE1	incised AP-DNA	PARP1	1.5	+0.04
FAM-APE1	gap-DNA	PARP1	1.2	+0.02
FAM-APE1	nick-DNA	PARP1	2.0	+0.04
FAM-Polβ	incised AP-DNA	XRCC1	1.1^d^	+0.16
FAM-Polβ	gap-DNA	XRCC1	1.0^d^	+0.19
FAM-Polβ	nick-DNA	XRCC1	1.0^d^	+0.18
FAM-Polβ	incised AP-DNA	PARP1	1.1	−0.04
FAM-Polβ	gap-DNA	PARP1	0.97	+0.04
FAM-Polβ	nick-DNA	PARP1	1.0	+0.01
FAM-PARP1	gap-DNA	Polβ	1.0	+0.17
FAM-PARP1	nick-DNA	Polβ	1.1	+0.09
FAM-Polβ	incised AP-DNA	TDP1	1.1	−0.04
FAM-Polβ	gap-DNA	TDP1	0.92	−0.05
FAM-Polβ	nick-DNA	TDP1	1.0	−0.08
FAM-PARP1	gap-DNA	XRCC1	0.95	+0.03
FAM-PARP1	nick-DNA	XRCC1	0.98	+0.05

^a^Titration experiments were performed at constant concentrations of the FAM-labeled protein (40 nM) and DNA (160 nM).

^b^Effect on the protein–protein binding affinity determined as ratio between the EC_50_ values in the presence and absence of DNA. The EC_50_ values in the presence of DNA are presented in Supplementary Table S5.

^c^Increase (+) or decrease (−) in the efficiency of FRET between the FAM- and TMR-labeled proteins in the presence of DNA.

^d^Binding affinities in the presence and absence of DNA were determined for the TMR-labeled protein partner.

The interaction between Polβ and XRCC1 was shown to be modulated by various model DNAs. Incised AP-DNA, gap-DNA and nick-DNA induced significant increase (by 16–19%) in the efficiency of FRET in the FAM-Polβ·TMR-XRCC1 complex, while having no influence on the protein–protein binding affinity. The intensity of FAM-XRCC1 fluorescence increased significantly upon binding to Polβ only in the presence of gap-DNA, enabling us to determine the EC_50_ value (as described above). The DNA-induced effects on the FRET efficiency in the FAM-XRCC1-TMR-Polβ pair were opposite to those in the inverse pair (a 7–10% decrease in the presence of gap-DNA or nick-DNA; data not shown). Thus, the conformational rearrangement of the protein–protein complex induced by DNA binding has different effects on the relative orientation of the donor and acceptor probes depending on their spatial location in the complex. A similar observation was obtained when binding of FAM-Polβ to PARP1 or of FAM-PARP1 to Polβ was probed in the absence or presence of DNAs. The efficiency of FRET changed more significantly in the FAM-PARP1-TMR-Polβ pair than in the inverse pair (Table 3). Despite of the DNA-induced rearrangement of the Polβ·PARP1 complex, the protein–protein binding affinity was practically unaffected by the presence of three different DNAs. Modulation of the FAM-Polβ interaction with TDP1 in the presence of DNA substrates of Polβ, as reflected by a slightly decreased (by 4–8%) efficiency of FRET, also had no appreciable effect on the binding affinity constant. Thus, the strength of Polβ interaction with regulatory and accessory proteins of BER is modulated by DNA intermediates to a lesser extent as compared to its interaction with APE1.

The interaction of XRCC1 with PARP1 and TDP1 was probed in the absence and presence of gap-DNA and nick-DNA. The experiments revealed no appreciable effects of canonical BER intermediates of the XRCC1 interaction with TDP1 (data not shown). An increased efficiency of FRET in the FAM-PARP1·XRCC1 complex detected in the presence of DNAs indicated the complex rearrangement, which nevertheless did not appreciably affect the protein–protein binding affinity (Table 3).

Analysis of the effects detected for various complexes revealed no relationship between the strength of protein–protein interaction and the extent of DNA-induced modulation of the complex. The most pronounced changes in the efficiency of FRET reflecting the DNA-induced rearrangements were detected for the FAM-Polβ·XRCC1 and FAM-PARP1·Polβ complexes characterized by quite different affinities. The greatest impacts of DNAs on the protein–protein binding affinity revealed for the APE1 complexes with various proteins were accompanied only by slight changes in the efficiency of FRET. It is conceivable that the ternary complexes are stabilized by direct protein–protein and DNA-mediated interactions whose relative contribution is specific for each complex.

### Analysis of protein–protein complexes by light scattering

**Figure 5.**
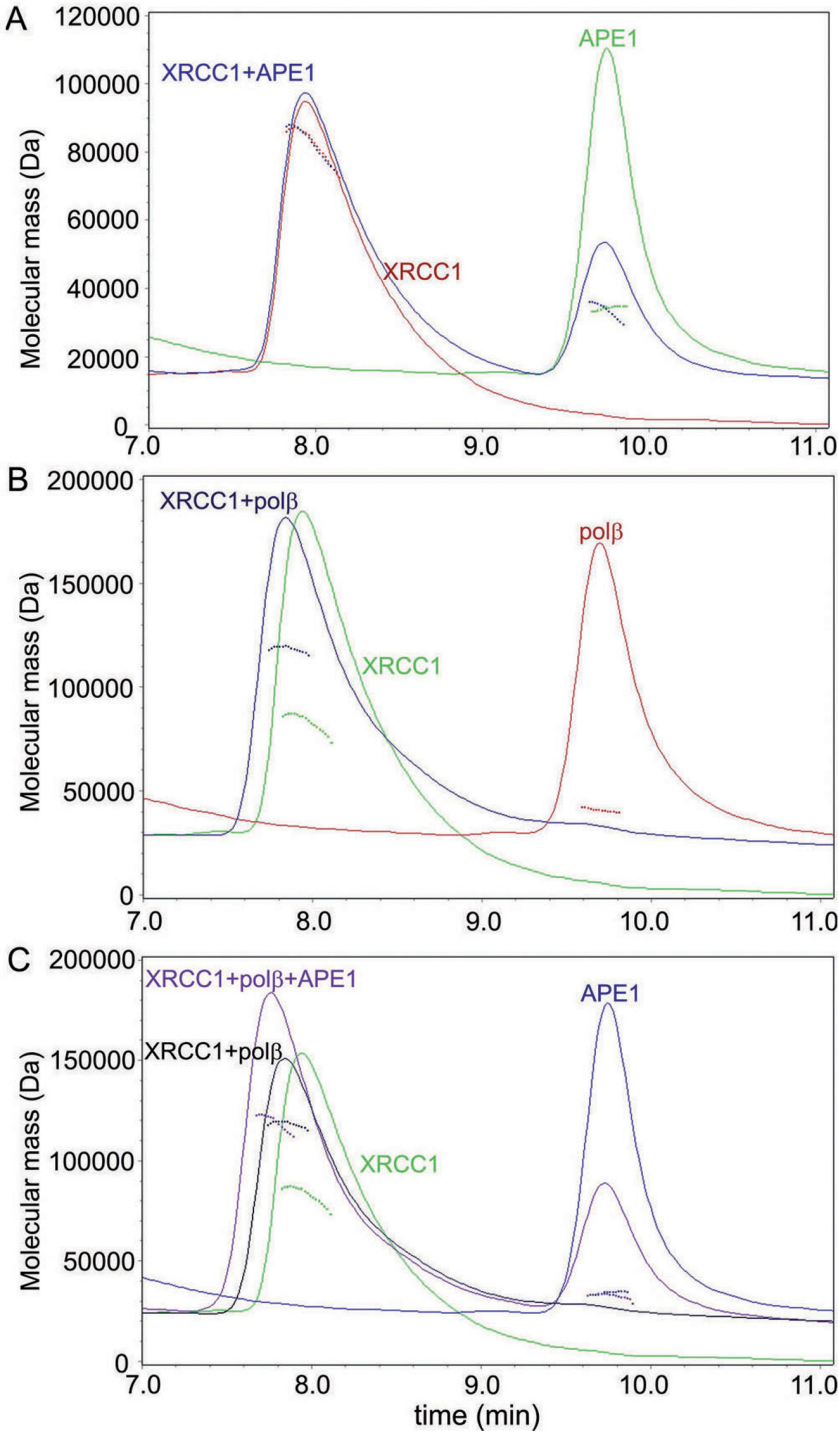
Analysis of protein oligomerization by SEC-MALLS experiments. (**A**) Overlay of chromatograms of XRCC1 (red), APE1 (green) and their equimolar mixture (blue). (**B**) Analysis of Polβ binding to XRCC1. Chromatograms of XRCC1, Polβ and their equimolar mixture are shown by green, red and blue traces respectively. (**C**) Overlay of chromatograms of XRCC1 (green), APE1 (blue) and equimolar mixtures of two (XRCC1 + Polβ, black) or three proteins (XRCC1 + Polβ + APE1, violet). The solid lines trace the absorbance at 280 nm of the eluate from a BioSep-SEC-S 3000 column as a function of time; the dotted lines represent the weight-average molecular weight of the species calculated from refractive index and light-scattering measurements.

To compare the stability of different homo- and hetero-oligomeric protein–protein complexes by alternative approaches, size exclusion chromatography coupled with ‘on-line’ multi-angle laser light-scattering (SEC-MALLS) was used. In these experiments, samples were fractionated on a gel-filtration column, and the absorbance at 280 nm and the refractive index of the eluate were monitored together with the multi-angle laser light scattering of the sample, allowing calculation of the weight-average molecular weight (Mw) of species across elution. We have analyzed samples of individual proteins (XRCC1, Polβ and APE1) and their equimolar mixtures (XRCC1 + Polβ, XRCC1 + APE1, Polβ + APE1, and XRCC1 + Polβ + APE1). The chromatograms presented in Figure 5 show that the individual proteins eluted from the SEC column as single peaks. The Mw values of XRCC1, Polβ and APE1 (Supplementary Table S6) determined by MALLS (∼83, ∼41 and ∼34 kDa respectively) correlate with those expected for monomeric forms (69.5, 38.3 and 35.6 kDa respectively). The highest discrepancy between the predicted and the experimentally determined Mw values observed for XRCC1 (19%) could be due to the non-globular shape of this protein. The equimolar mixture of Polβ and APE1 eluted as a single peak (data not shown) associated with the Mw value (∼35 kDa), which was between those for Polβ and APE1 (Supplementary Table S6), and clearly resulted from their co-elution. The equimolar mixture of XRCC1 and APE1 was resolved on the column as two species with Mw values and retention times closely similar or identical to those of the individual components (Figure 5A and Supplementary Table S6). Separation of a mixture of XRCC1 and Polβ at a 1:1 molar ratio produced a new species with a Mw value ∼118 kDa, which corresponds most closely to the Mw predicted for the heterodimeric XRCC1·Polβ complex (107.8 kDa; Figure 5B and Supplementary Table S6). The relative absorbance at 280 nm at the trailing edge of the peak is significantly higher in the heterocomplex than in XRCC1, indicating partial dissociation of the complex during SEC. Thus, only one of six binary protein–protein complexes (homo- and hetero-oligomeric) detected by fluorescence titration experiments was stable under the SEC-MALLS experiments. The XRCC1·Polβ complex is characterized by the highest binding affinity as shown by comparison of the respective EC_50_ values (Table 1). Attempts were made to detect a ternary complex of XRCC1 with Polβ and APE1. The elution profile of the equimolar mixture of the three proteins showed two major species (Figure 5C). The first peak was associated with a Mw value of 119 kDa, that only slightly exceeded the Mw of the XRCC1·Polβ complex, but it was obviously less retarded on the SEC column when compared to the binary complex. The second peak overlapped well with the APE1 peak. The data suggest the formation of the ternary XRCC1·Polβ·APE1 complex, which is less stable than the binary XRCC1·Polβ complex and more stable than the binary complexes of APE1 with XRCC1 or Polβ (not detected by SEC-MALLS under identical experimental conditions).

To determine the oligomeric state of proteins and of protein–protein complexes in solution, we analyzed them by dynamic light scattering (DLS). DLS processes the time-dependent fluctuations in scattered light to yield the hydrodynamic radius (*R*_H_) of diffusing particles in solution under true equilibrium (31). The average *R*_H_ values were measured for individual Polβ, APE1, XRCC1, and their equimolar mixtures (Supplementary Table S7). The protein concentrations exceeded the apparent equilibrium dissociation constants by ≥30-fold, thus ensuring a high extent of oligomerization. In each case, the size distribution profile as a function of the intensity revealed a single peak, indicating that the protein solution was homogeneous (data not shown). The *R*_H_ values determined for Polβ, APE1 and their complex sufficiently agreed with those predicted for the respective homodimers and heterodimer. This indicated that the main oligomeric state of homo- and hetero-associated complexes of Polβ and APE1 was dimeric. The *R*_H_ value determined for XRCC1 significantly exceeded the predicted value for the homodimer (5.8 versus 4.9 nm). The discrepancy obviously results from the non-spherical shape of XRCC1. Based on sedimentation equilibrium data, XRCC1 was proposed to be a prolate ellipsoid (with an axial ratio of about 8:1) existing in a monomer-dimer equilibrium (29). Using the DTSv612 software (Malvern Instruments Ltd.), we estimated the *R*_H_ values for the XRCC1 monomer and dimer, based on the ellipsoidal model. The *R*_H_ values were calculated to be 4.8 nm for the monomer, 6.0 nm for the dimer with the axial ratio of 8:1 and 7.6 nm for the dimer with the axial ratio of 16:1. Their comparison with the experimentally determined *R*_H_ values (Supplementary Table S7) indicated that XRCC1 was mostly dimerized. The *R*_H_ values measured for the XRCC1·Polβ complex (from the intensity and volume size distributions) were closely similar to those of XRCC1. These results indicated a predominant contribution of the homodimeric XRCC1 molecule to the size and shape of the heterocomplex. The most likely oligomeric state of the XRCC1·Polβ complex, that combines self-association and hetero-association of XRCC1, is a heterotetramer. The XRCC1·Polβ heterodimer detected by SEC-MALLS could result from shifting a heterotetramer-heterodimer equilibrium to the most stable form during chromatographic separation. The *R*_H_ value of the XRCC1·APE1 complex was shown to be significantly (∼1.3-fold) smaller than those of the XRCC1·Polβ complex and the XRCC1 homodimer, and closer to the *R*_H_ value calculated for the XRCC1 monomer (based on the ellipsoidal model). These data suggested that the main form of the XRCC1·APE1 complex was heterodimeric. The *R*_H_ value measured for the triple protein mixture (XRCC1 with Polβ and APE1) was between those for the binary complexes of XRCC1 with Polβ or APE1. The data did not exclude coexistence of the ternary XRCC1·Polβ·APE1 complex with the most stable XRCC1·Polβ complex and other species, and could not be interpreted unambiguously.

## DISCUSSION

BER is an exceptionally efficient process that protects mammalian cells against the most common DNA lesions usually limited to single nucleotide modifications. Each of the two BER sub-pathways (SP and LP) involves multiple protein–protein interactions facilitating the accumulation of proteins at sites of chromosomal DNA damage and coordination of the sequential enzymatic steps. Most of the previously identified complexes between BER components have been detected by biochemical and immunological techniques that are unsuitable to quantify the affinity and dynamics of macromolecular interactions. Such information is however critical for a better understanding of the organization and regulation of BER. In this study, a set of biophysical approaches was employed to quantitatively characterize interactions between BER proteins.

Steady-state fluorescence titration experiments were performed to determine the relative binding affinities of APE1, Polβ, PARP1 and XRCC1 for various protein partners (Figures 1–3). The fully active labeled proteins were prepared by covalent attachment of a fluorescent probe (fluorescein as a donor, and tetramethylrhodamine as an acceptor) under conditions optimal for primary labeling of the terminal amino group. We succeeded in detecting and characterizing fourteen homo- and hetero-oligomeric complexes formed by individual full-length proteins involved in BER (Table 1). Physical interaction between the proteins in most of the complexes was demonstrated by FRET experiments (Table 2). The fluorescence-based approaches allowed us to quantify the effects of BER intermediates on the protein–protein interactions (Table 3).

Direct interaction of APE1 with PARP1 and TDP1, of Polβ with TDP1, and self-association of APE1 have never been detected to date (Supplementary Table S1). APE1 and PARP1 are components of multi-protein complexes proposed to be involved in BER at higher levels of DNA damage and in a DNA replication-associated BER (32). It was shown that APE1 and PARP1 can interact with the same BER intermediates, and competition between these enzymes modulates the functions of each partner (9,10,30,33). Opposite effects of PARP1 on the 3′-exonuclease activity of APE1 were observed at low and high enzyme concentrations, indicating that differences in the stoichiometry of the BER enzymes may regulate BER (10). We found here that the strength of the interaction between APE1 and PARP1 is modulated to a different extent by various BER intermediates (Table 3). Evidently, interaction between APE1 and PARP1 contributes to regulation of their functions in BER, together with the competition of APE1 and PARP1 for binding to DNA ligands. APE1 and PARP1 are abundant nuclear proteins [reviewed in (10)], and their interaction *in vivo* is highly probable.

TDP1 was found in a multi-protein complex with PNKP, XRCC1, PARP1 and LigIIIα associated with FLAG-tagged Polβ (34). However, interaction of TDP1 with Polβ could be mediated by other components of the complex, such as PARP1, XRCC1 and LigIIIα, shown by independent approaches to interact with TDP1 (35–37). Indeed, we have detected the ternary complex Polβ·TDP1·XRCC1 by titration of FAM-Polβ with XRCC1 in the presence of TDP1 (Figure 3B). TDP1 promotes the repair of topoisomerase I-mediated DNA damage and AP/3′-dRP lesions via its tyrosyl-DNA phosphodiesterase and AP/3′-dRP lyase activities, and is implicated in processing various types of blocking 3′-lesions as a general 3′-phosphodiesterase [reviewed in (38)]. Polβ and TDP1 are involved in repair of AP sites when their cleavage is initiated by TDP1 (13). However, as yet no direct evidence indicating functional coupling between the enzymatic activities of TDP1 and Polβ has been reported. There are no data demonstrating interplay between APE1 and TDP1. Further studies are required to understand the functional significance of the TDP1 interaction with Polβ and APE1.

Interaction between two major BER enzymes, APE1 and Polβ, extensively studied by different approaches was not detected when the individual proteins were used (39,40). Their specific interaction demonstrated by yeast two-hybrid analyses could be mediated by additional component(s) of cell extracts (39). Ternary APE1·Polβ·DNA complexes formed at different BER intermediates were detected by gel mobility shift assays (26,39). Coupling of enzymatic activities of the multifunctional APE1 (41) and the bifunctional Polβ (42) was demonstrated in many reports. APE1 stimulates the nucleotidyl-transferase and dRP-lyase activities of Polβ (10,26,39). Polβ inhibits the 3′-exonuclease activity of APE1 when the enzyme concentrations are comparable, whereas excess APE1 over Polβ allows APE1 to perform the proofreading function (10). It was suggested that step-by-step coordination in SP BER can rely on DNA binding specificity inherent in APE1 and Polβ and on APE1·Polβ·DNA complex formation (2,26). The interaction of APE1 and Polβ with the APE1-incised BER intermediate in cell extracts of mouse embryonic fibroblasts was determined by the photoaffinity labeling technique (9). For the first time here, we quantitatively characterized the interaction between APE1 and Polβ in the absence and presence of various BER intermediates under true equilibrium conditions, using fluorescence-based approaches. Distinct effects were produced by the various DNAs on the APE1-Polβ binding affinity and FRET efficiency (Table 3), indicating that they are related to regulation and coordination of the enzymatic functions during BER. An unexpected finding is that Polβ interacts more strongly with APE1 in the presence of AP site-containing DNA than in the complex mimicking a step after the APE1-catalyzed incision. This result suggests that the APE1-incised BER intermediate is effectively channeled to Polβ immediately during the incision step. The efficiency of FRET within the FAM-APE1·TMR-Polβ complex appeared sensitive to the presence of XRCC1 and PARP1 (Table 2), indicating modulation of the APE1-Polβ interaction induced by the formation of ternary protein–protein complexes. The coordination of enzymatic steps mediated by APE1 and Polβ in XRCC1- and PARP1-dependent BER sub-pathways is obviously facilitated by interactions of the enzymes with each other and with the regulatory proteins. The relative binding affinities of PARP1 for APE1 and Polβ in the presence of incised AP-DNA or nick-DNA are appreciably higher as compared to those of Polβ for APE1 (Supplementary Table S5). These results provide an explanation for the inhibiting effect of PARP1 on the APE1-induced stimulation of LP BER strand-displacement synthesis catalyzed by Polβ (10). Thus, one of the regulatory mechanisms of BER is modulation of the strength of interactions between the enzymes and accessory proteins by the DNA intermediates.

Most interacting partners of PARP1 detected here by fluorescence titration (with the exception of APE1, see discussion above) are known from previous reports (Supplementary Table S1) but their interactions have not been yet characterized quantitatively. Apparent dissociation constants determined for homo- and hetero-oligomeric complexes of PARP1 with Polβ, XRCC1 and TDP1 (EC_50_ values in Table 1) are closely similar. We compared the binding affinities of different proteins for the full-length PARP1 and its N-terminal 24-kDa apoptotic fragment (p24) composed of two zinc-finger subdomains of the DNA binding domain (DBD). The FAM-labeled APE1, Polβ and PARP1 bind PARP1 with a significantly higher affinity than the p24 protein, indicating that the BRCT-domain known as the second protein–protein interaction domain in PARP1, in addition to the DNA-binding domain (DBD; Supplementary Table S1), contributes for the most part to PARP1 association with various proteins. The binding affinity of PARP1 for Polβ and XRCC1 did not appear to be modulated by canonical BER intermediates (Table 3). One way to enhance the specificity of PARP1 interaction with proteins is posttranslational modification of proteins with poly(ADP-ribose) (PAR). The PAR-binding sequence motif overlapping with domains responsible for protein–protein interactions was identified in several DNA damage checkpoint proteins (43). XRCC1 and LigIIIα were shown to associate preferentially with PARylated PARP1 (44,45), and PARylation-dependent accumulation of XRCC1 at sites of oxidative or UV-induced DNA damage was demonstrated (46,47). In a recent study of XRCC1 interaction with PARylated PARP1 by a quantitative fluorescence-based assay, it was found that XRCC1 binds selectively and with high affinity to PAR ligands longer than 7 ADP-ribose units in length (48). The lowest EC_50_ value determined for these XRCC1 complexes (17 nM) is significantly less in comparison with the respective value determined for the XRCC1·PARP1 complex (Table 1), explaining the preferential association of XRCC1 with PARylated PARP1. The ability of XRCC1 to interact with intact PARP1 in the presence of BER intermediates (Table 3) may contribute to the negative regulation of PARP1 auto-PARylation by an excess of XRCC1, detected *in vitro* and *in vivo* and proposed to protect DNA ends produced during DNA repair (44).

**Figure 6.**
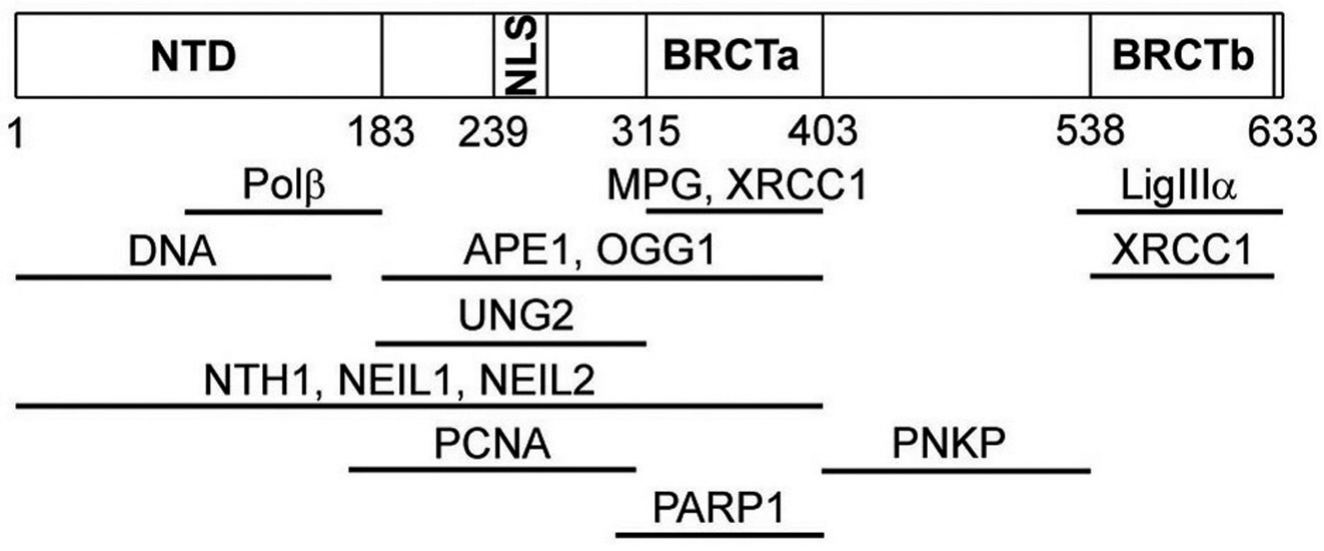
XRCC1 interactions with BER proteins. Domain architecture of human XRCC1 and distinct sites of interactions with itself and other proteins (summarized in Supplementary Table S1) are schematically shown.

Direct interaction of XRCC1 with multiple proteins was shown previously, and for most partners specific sites were identified (Figure 6 and Supplementary Table S1). Limited quantitative data were obtained by several approaches that included equilibrium and non-equilibrium techniques (4,49–53). Most of them characterize interactions between individual protein domains. We have determined the apparent dissociation constants of homo- and several hetero-oligomeric XRCC1 complexes formed by the full-length proteins (Table 1). The EC_50_ values can be considered as relative binding affinity constants assayed by an equilibrium technique under identical conditions. The lowest *K*_d_ was determined for the XRCC1 complex with Polβ. It should be noted that this value is lower than the concentration of the labeled protein and therefore represents the upper limit for the *K*_d_ of this complex. Further experiments performed by a non-equilibrium SEC-MALLS technique confirmed the superior stability of the XRCC1·Polβ complex as compared to other homo- and hetero-oligomeric complexes formed by XRCC1, Polβ and APE1. Based on the results of SEC-MALLS and DLS measurements of molecular size (Supplementary Tables S6 and S7), we propose that Polβ forms a permanent heterodimeric complex with XRCC1 (Supplementary Figure S3), existing in equilibrium with a less stable heterotetramer formed via XRCC1 homo-oligomerization. The interaction between Polβ and XRCC1 is required to recruit Polβ to sites of DNA damage and for efficient BER (5,32,54–57). A recent study has revealed that the primary function of this interaction is to maintain stability of each protein by preventing proteasome-mediated degradation (58). Previously, a constitutive complex of XRCC1 with LigIIIα was proposed to be required to stabilize the cellular level of LigIIIα (59). The *K*_d_ values determined by gel-filtration in studies of the domain specific interaction in the XRCC1·Polβ (50) and XRCC1·LigIIIα complexes (4) are nearly comparable (300 nM versus 100 nM), and significantly exceed the *K*_d_ of the XRCC1·Polβ complex determined in the present study. The XRCC1 domains responsible for the interaction with Polβ and LigIIIα are distinct and separate (Figure 6 and Supplementary Table S1), thus allowing the formation of a permanent Polβ·XRCC1·LigIIIα complex (Supplementary Figure S3). Moreover, the XRCC1 binding sites for Polβ and LigIIIα do not overlap with regions mediating interactions with most other protein partners, thus enabling participation of the preformed ternary complex in the entire Polβ- and XRCC1-dependent BER sub-pathway. We propose that the preformed Polβ·XRCC1·LigIIIα complex serves as a starting point for a dynamic assembly and disassembly of higher order complexes involved in recognition and processing of different types of DNA damage by SP BER. Polβ and LigIIIα accumulate at the damage sites synchronously with XRCC1, in contrast to other XRCC1 interacting proteins (57), suggesting the formation of permanent Polβ·XRCC1·LigIIIα complex *in vivo*. We revealed DNA-induced changes in the efficiency of FRET in the FAM-XRCC1·TMR-Polβ complex, that were most pronounced among the effects detected for various XRCC1 complexes (Table 3). Such differences can be explained by the fact that the N-terminal domain (NTD) of XRCC1 bearing the fluorescent label mediates the XRCC1 interaction with both Polβ and DNA (Figure 6). The rearrangement of the XRCC1·Polβ complex induced by various BER intermediates was not accompanied by a detectable change in protein–protein binding affinity (Table 3). The stability of the protein–protein interaction in the DNA-bound state is particularly important for the functioning of XRCC1 as the scaffold protein in the Polβ/XRCC1 coordinated BER.

## SUPPLEMENTARY DATA

Supplementary Data are available at NAR Online.

SUPPLEMENTARY DATA
