# Supplementary material for: Quantitative characterization of protein–protein complexes involved in base excision DNA repair
Source: Nucleic Acids Res. 2015 May 26;43(12):6009–22. doi: 10.1093/nar/gkv569 (PMC4499159; doi:10.1093/nar/gkv569)
Supplement: SUPPLEMENTARY DATA [file supp_43_12_6009__index.html]

Quantitative characterization of protein–protein complexes involved in base excision DNA repair — SUPPLEMENTARY DATA 

# Quantitative characterization of protein–protein complexes involved in base excision DNA repair

## SUPPLEMENTARY DATA

- SUPPLEMENTARY DATA
