# Supplementary material for: Quantitative characterization of protein–protein complexes involved in base excision DNA repair
Source: Nucleic Acids Res. 2015 May 26;43(12):6009–22. doi: 10.1093/nar/gkv569 (PMC4499159; doi:10.1093/nar/gkv569)
Supplement: SUPPLEMENTARY DATA [file supp_gkv569_nar-03572-d-2014-File008.pdf]

SUPPLEMENTARY DATA

Quantitative characterization of protein-protein complexes involved in base excision DNA repair

Nina A. Moor[1], Inna A. Vasil'eva[1], Rashid O. Anarbaev[1], Alfred A. Antson[2] and Olga I. Lavrik[1,3]*

[1]Institute of Chemical Biology and Fundamental Medicine, Siberian Branch of the Russian Academy of Sciences, Novosibirsk, 630090, Russia
[2]Structural Biology Laboratory, Department of Chemistry, University of York, York YO10 5DD, UK
[3]Department of Natural Sciences, Novosibirsk State University, Novosibirsk, 630090, Russia

*To whom correspondence should be addressed. Tel: +7 383 363 5195; Fax: +7 383 363 5153; Email: lavrik@niboch.nsc.ru

The authors wish it to be known that, in their opinion, the first two authors should be regarded as joint First Authors.

SUPPLEMENTARY INTRODUCTION

Table S1. Interactions between proteins involved in Base Excision Repair

Protein, domain[a]	Interacting partner (domain)[a]	References
XRCC1, NTD	Polβ (CD); DNA substrates	(1–4)
XRCC1, NLS	PCNA; UNG2 (CD)	(5,6)
XRCC1, NTD + NLS	NTH1 (CTD); NEIL1 (CTD); NEIL2 (NTD)	(7–9)
XRCC1, NLS + BRCTa	APE1; OGG1	(10)
XRCC1, BRCTa	XRCC1; PARP1 (DBD, BRCT); PARP2 (ED); MPG; NTH1 (CTD); NEIL1 (CTD); NEIL2 (NTD)	(7–9,11–14)
XRCC1, linker 404-537	PNKP (NTD/CD)	(15,16)
XRCC1, BRCTb	XRCC1; LigIIIα (BRCT)	(10,17,18)
XRCC1	TDP1; aprataxin	(19,20)
PARP1, DBD + BRCT	Polβ (CD); PARP1; PARP2; LigIIIα (55-122)	(14,21,22)
PARP1, CD	TDP1 (NTD, 1-185)	(23)
Polβ, CD	Polβ; PARP2 (ED)	(14,24)
Polβ	PNKP; APE1	(25–27)
Polβ, NTD	NEIL1 (CTD); NEIL2 (NTD); LigI (NTD)	(8,9,24)
LigIIIα, BRCT	LigIIIα (BRCT); NEIL1 (CTD); NEIL2 (NTD); PARP2 (ED); TDP1 (NTD)	(8,9,13,14, 28)
LigIIIα	PNKP	(25)
LigI	LigI	(24)

[a]Domain comprising the interacting region is indicated (when identified).
Domain composition of proteins: human XRCC1 (633 aa): NTD 1-183, NLS 239-266, BRCTa 315-403, BRCTb 538-629 (29); human PARP1 (1014 aa): ZnF1 1-96, ZnF2 97-206, NLS 207-240, ZnF3 241-366, BRCT 381-484, WGR 518-661, CD 662-1014 (30); murine PARP2 (559 aa): NTD 1-63, ED 64-198, CD 199-559 (14); human (rat) Polβ (335 aa): NTD 1-87, CD 88-335 (fingers 88-151, palm 152-262, thumb 263-335) (31); human LigIIIα (922 aa): ZnF 1-100, linker region 101-170, DBD 171-390, CD 391-836, BRCT 837-922 (32); human PNKP (521 aa): NTD 1-110, CD 146-521 (33). Domain designation: CD – catalytic domain; CTD – C-terminal domain; DBD – DNA binding domain; NLS – nuclear localization signal; NTD – N-terminal domain; ZnF – zinc-finger domain.

SUPPLEMENTARY MATERIALS AND METHODS

Enzyme activity assays

The enzymatic activity of Polβ in DNA synthesis was verified on activated DNA. The reaction mixture contained 50 mM Tris-HCl, pH 8.0, 50 mM NaCl, 10 mM $MgCl_2$, 5 mM DTT, 10 μM dNTP (dATP, dGTP, dTTP), 2 μM α-[^{32}P]dCTP, and 1 A_{260}/ml of activated DNA (high-molecular mass DNA treated with DNAse I). The reaction was initiated by adding Polβ, unlabeled or labeled with a dye (TAF, FAM or TMR), to a final concentration 250–1000 nM. The reaction mixtures were incubated at 37°C for 1–10 min. The amount of ^{32}P-labeled DNA in aliquots was determined by trichloroacetic acid precipitation as described (34).

The endonuclease activity of APE1 was assayed in reaction mixtures containing 50 mM Tris-HCl, pH 8.0, 50 mM NaCl, 10 mM $MgCl_2$, 5 mM DTT and 10 nM 5'-[^{32}P]-labeled DNA substrate. The natural AP site in the DNA substrate was prepared by treatment of U-containing 32-base pair oligonucleotide with UDG (0.5 U/ml) for 15 min at 37°C immediately before the experiments. After adding 1–10 nM APE1, unlabeled or labeled with a dye (TAF, FAM or TMR), the reaction mixtures were incubated at 37°C for 1–10 min. Aliquots were further incubated with 20 nM $NaBH_4$ for 30 min at 0–1°C (on ice). Reaction products were separated by electrophoresis in 10% denaturing polyacrylamide gels. The extent of DNA cleavage at the AP-site was quantified by Phosphor imaging.

Preparation of DNA ligands for binding experiments

DNA oligonucleotides were synthesized and purified in the Laboratory of Medicinal Chemistry, Institute of Chemical Biology and Fundamental Medicine, Novosibirsk, Russia; pUC19 DNA was from SibEnzyme LTD, Novosibirsk, Russia. Sequences of oligonucleotides were as follows:
template – 5’-GGAAGACCCTGACGTTACCCAACTTAATCGCC-3’,
complementary oligonucleotide 1 – 5’-GGCGATTAAGTTGGGTAACGTCAGGGTCTTCC-3’
and oligonucleotide 2 – 5’-GGCGATTAAGTTGGGFAACGTCAGGGTCTTCC-3’ (F = 3-hydroxy-2-hydroxymethyltetrahydrofuran, THF);
upstream primer 1 – 5’-GGCGATTAAGTTGGG-3’ and primer 2 – 5’-GGCGATTAAGTTGGGT-3’,
downstream primer 1 – 5’-pAACGTCAGGGTCTTCC-3’ (5’-p indicates the presence of a phosphate at the terminus) and primer 2 – 5’-pFAACGTCAGGGTCTTCC-3’.

32-mer double stranded (ds) oligonucleotides containing a one-nucleotide gap (gap-DNA) or nick (nick-DNA) were prepared by annealing the upstream primer 1 or 2 respectively and the downstream primer 1 to the template oligonucleotide mixed in equimolar ratios. The mixture was heated at 90°C for 2 min, and then slowly cooled down to room temperature. A non-gapped 32-mer DNA (ds-DNA) and a 32-mer DNA substrate with an abasic site (AP-DNA) were prepared by annealing the complementary oligonucleotide 1 or the THF-containing oligonucleotide 2 to the template. A 32-mer 1-nt-gapped DNA with a 5’-pTHF group (incised AP-DNA) was prepared by annealing the upstream primer 1 and the downstream primer 2 to the template. The amount of duplex DNA was controlled by native 10% polyacrylamide gel electrophoresis. A nicked pUC19 DNA substrate (nick-pUC19) was prepared by

treatment of pUC19 DNA with site-specific *Bacillus stearothermophilus* nickase N.Bst9I (SibEnzyme LTD, Novosibirsk) according to the recommended protocol. The reaction mixture contained 10 mM Tris-HCl, pH 8.5, 150 mM KCl, 10 mM $MgCl_2$, 1 mM DTT, 140 µg/ml pUC19 DNA and 175 U/ml nickase N.Bst9I. The reaction was performed for 2 h at 55°C, and then stopped by extracting the enzyme with a phenol-chloroform mixture. The nicked pUC19 DNA was precipitated with ethanol and washed with 70% ethanol. The amount of relaxed form of pUC19 DNA was controlled by electrophoresis in 1% agarose (data not shown).

Fluorescence studies of protein-DNA interactions

Binding of BER proteins to DNA ligands was examined by fluorescence titration experiments. Fluorescence intensities of solutions of the TAF(FAM)-labeled protein (at a fixed concentration) in binding buffer were measured in the absence and presence of various concentrations of the DNA ligand. The binding buffer contained 50 mM HEPES, pH 8.0, 100 mM NaCl and 4 mM DTT. Titration of the TAF(FAM)-labeled APE1 by various DNAs was performed in binding buffer supplemented with 10 mM EDTA (to suppress the endonuclease and the 3'-exonuclease activities of APE1). All other details of the measurements were the same as in fluorescence studies of protein-protein interactions described in the Materials and Methods section of the main text.

SUPPLEMENTARY RESULTS

Synthesis and characterization of fluorescent-labeled proteins

To prepare functionally active labeled proteins, the reaction conditions were optimized by varying the molar excess of reagent over the protein, and the incubation time. The stoichiometry of labeling of APE1 and Polβ with different reagents in varying conditions was determined and the enzymatic activity of the labeled proteins was tested (Supplementary Table S2). The catalytic activity was retained when the stoichiometry of labeling of APE1 with any of the reagents used and that of Polβ with FAM-SE or TMR-SE did not exceed 1 mol of dye per mol of protein. The functional activity of Polβ was more sensitive to labeling with DTAF than that of APE1. An active TAF-labeled Polβ was prepared in conditions in which only half the protein was labeled. Thus, the succinimidyl esters are preferable for labeling to ensure functional activity of the dye-labeled proteins. Molar excess of the reactive probe over the protein in the range of 1.6–2-fold is optimal to prepare fully active FAM(TMR)-labeled APE1 and Polβ with the labeling stoichiometry of ~0.7–1 mol of dye per mol of protein. The loss of enzymatic activity observed when labeling of APE1 and Polβ was performed with a higher excess (4–10-fold) of either TMR-SE or FAM-SE is most likely due to acylation of amino acid residues (together with the terminal amino group) involved in the catalytic function of enzyme and/or DNA-substrate binding within the active site. The terminal amino group (which is the primary site of labeling at pH 7.0) of both APE1 and Polβ is not immediately involved in the enzymatic function (35,36). The optimal reaction conditions found for APE1 and Polβ were used to synthesize other dye-labeled proteins, namely PARP1, XRCC1 and TDP1 (Supplementary Table S2). In these conditions, the stoichiometry of PARP1 and TDP1 labeling with FAM-SE and/or TMR-SE falls within the same range as that of APE1 and Polβ. The stoichiometry of XRCC1 labeling with all three reactive probes did not exceed 0.45 mol of dye per mol of protein. When the reaction was performed with a higher reagent excess, part of the labeled protein precipitated irreversibly. Further optimization of the reaction conditions by varying the protein and reagent concentrations enabled us to achieve close to stoichiometric labeling of XRCC1 with TMR-SE (for FRET measurements).

Table S2. Labeling of APE1, Polβ, PARP1, XRCC1 and TDP1 with various reactive probes

Protein	Reagent	Molar ratio reagent:protein; incubation time[a]	Stoichiometry of labeling, mol of dye/mol of protein[b]	Activity, %[b,c]
Polβ	TMR-SE	1.6:1; 13 h	0.75 ± 0.03*	101± 4
Polβ	TMR-SE	4:1; 2 h	1.3 ± 0.1	80 ± 2
Polβ	TMR-SE	4:1; 5 h	1.7 ± 0.2	66 ± 3
Polβ	TMR-SE	10:1; 13 h	3.4 ± 0.2	3 ± 1
Polβ	FAM-SE	1.6:1; 13 h	0.76 ± 0.03*	98 ± 2
Polβ	DTAF	2:1; 17 h	0.45 ± 0.03	90 ± 4
Polβ	DTAF	3:1; 17 h	0.68 ± 0.03*	76 ± 3
APE1	TMR-SE	1.6:1; 13 h	0.86 ± 0.03*	99 ± 3
APE1	TMR-SE	2:1; 2 h	0.72 ± 0.04	101 ± 3
APE1	TMR-SE	2:1; 13 h	1.1 ± 0.05	95 ± 3
APE1	TMR-SE	10:1; 13 h	3.8 ± 0.2	4 ± 1
APE1	FAM-SE	1.6:1; 13 h	0.89 ± 0.04*	98 ± 2
APE1	DTAF	3:1; 17 h	0.86 ± 0.03*	98 ± 4
PARP1	FAM-SE	2:1; 13 h	0.84 ± 0.03*	
PARP1	TMR-SE	2:1; 13 h	0.79 ± 0.03*	
XRCC1	DTAF	3:1; 17 h	0.45 ± 0.04*	
XRCC1	FAM-SE	2:1; 13 h	0.43 ± 0.05*	
XRCC1	TMR-SE	2:1; 13 h	0.41 ± 0.04	
XRCC1[d]	TMR-SE	3:1; 13 h	0.84 ± 0.03*	
TDP1	TMR-SE	2:1; 13 h	0.79 ± 0.03*	

[a]The reaction mixture contained 100 mM MES, pH 7.0, 150 mM NaCl, 100 μM protein and varying concentrations of the reactive probe. TMR-SE, FAM-SE – N-succinimidyl ester of 5(6)-carboxytetramethylrhodamine or of 5(6)-carboxyfluorescein, DTAF – 5-(4,6-dichlorotriazinyl)aminofluorescein.

[b]Values are the mean (± s.d.) of three measurements on samples from the same preparation of protein (labeled Polβ, APE1 or PARP1) or from different preparations (labeled XRCC1). Labeling stoichiometry of proteins used in the titration and FRET experiments is asterisked.

[c]The catalytic activity of the dye-labeled enzyme is normalized to the activity of the respective unlabeled enzyme, measured in identical conditions as described in the Materials and Methods section (Supplementary Data).

[d]The labeling reaction was performed at 50 μM concentration of XRCC1.

Detection and quantification of protein-protein interactions by fluorescence titration experiments

Table S3. Parameters of protein-protein interactions determined by fluorescence titration

Labeled protein[a]	Protein partner	F_∞/F_0[b]	n[b]
FAM-APE1	APE1	2.2 ± 0.2	1.2 ± 0.2
FAM-APE1	Polβ	2.8 ± 0.2	1.9 ± 0.2
FAM-APE1	XRCC1	1.9 ± 0.2	1.6 ± 0.3
FAM-APE1	PARP1	2.4 ± 0.2	1.6 ± 0.3
FAM-APE1	p24	2.5 ± 0.4	1.2 ± 0.2
FAM-APE1	TDP1	2.4 ± 0.3	1.8 ± 0.2
FAM-Polβ	Polβ	2.7 ± 0.3	2.4 ± 0.4
FAM-Polβ	PARP1	2.4 ± 0.3	2.4 ± 0.4
FAM-Polβ	p24	1.7 ± 0.2	1.4 ± 0.2
FAM-Polβ	TDP1	2.2 ± 0.2	2.3 ± 0.3
FAM-PARP1	PARP1	2.9 ± 0.2	1.3 ± 0.2
FAM-PARP1	p24	2.3 ± 0.3	1.3 ± 0.1
FAM-PARP1	Polβ	2.6 ± 0.2	2.4 ± 0.2
FAM-PARP1	XRCC1	1.8 ± 0.1	2.4 ± 0.2
FAM-PARP1	TDP1	2.4 ± 0.2	2.0 ± 0.3
TAF-XRCC1	XRCC1	1.6 ± 0.2	1.9 ± 0.3
TAF-XRCC1	Polβ	1.4 ± 0.1	1.2 ± 0.2
FAM-XRCC1 + gap-DNA	Polβ	1.5 ± 0.2	2.2 ± 0.4
FAM-XRCC1	TDP1	1.4 ± 0.2	1.7 ± 0.3

[a]Titration experiments were performed at a constant concentration of the fluorescein-labeled protein (40 nM); gap-DNA (a 32-mer 1-nt-gapped DNA shown in Supplementary Figure S1) was added at a 3-fold molar excess over the FAM-XRCC1.

[b]Parameters derived from the titration curves by fitting to the four-parameter equation; F_∞/F_0 is the extent of change in the fluorescence intensity at saturation; n is the Hill coefficient. Values are the mean (± s.d.) of at least three independent experiments.

Fluorescence titration studies of protein-DNA interactions

To compare the affinities of BER proteins for each other and for various DNA ligands, the binding of four fluorescein-labeled proteins (Polβ, APE1, XRCC1 and PARP1) to DNA structures (Supplementary Figure S1) mimicking intermediates of DNA repair at different steps was examined. A synthetic 1-nt-gapped DNA (gap-DNA) is a canonical substrate of Polβ in SP BER. A nicked DNA with an internal 5'-phosphate (nick-DNA) and a nicked pUC19 (nick-pUC19) imitate products of the Polβ-catalyzed reaction, which can be processed further by a ligase activity or can serve as Polβ substrates in LP BER. A double-stranded DNA with a synthetic abasic site (AP-DNA) is an initial BER substrate of APE1. The double-stranded DNA (ds-DNA) and plasmid DNA are control DNAs containing no damage (mimicking the final product of DNA repair or the undamaged DNA). Typical titration curves obtained in experiments with three TAF-labeled proteins and gap-DNA are shown in Supplementary Figure S2. Binding parameters determined from the titration experiments are summarized in Supplementary Table S4. The fluorescence intensity of proteins increased at sub-saturating concentrations of DNA ligands to different extents, depending on the protein-DNA pair and the type of fluorescent label. Titration of FAM-labeled APE1 and PARP1 with DNA ligands revealed a slight increase in the fluorescence intensity that was insufficient to allow us to determine the binding parameters. The fluorescence intensity of FAM-XRCC1 appeared to be insensitive to binding of synthetic DNAs (data not shown). The experiments revealed that Polβ, APE1 and XRCC1 bind preferably damaged DNAs (BER intermediates). The apparent dissociation constants (EC_{50} values) of their complexes with the 1-nt-gapped DNA (the canonical substrate of Polβ) were comparable. XRCC1 displayed a 3-fold higher affinity for the nick-pUC19 as compared to that of Polβ. XRCC1 and APE1 bound the double-stranded DNA with a 1.6-fold higher affinity than Polβ. Both Polβ and XRCC1 bound the synthetic DNA duplex with one order higher affinity than the plasmid DNA pUC19. Clearly, DNA supercoiling affects interaction of BER proteins with DNA. It should be noted, that the Hill coefficient determined in experiments with the plasmid pUC19 and with nick-pUC19 was substantially less than one, suggesting existence of a few binding sites for proteins. A comparison of the binding parameters determined for the protein-protein and protein-DNA complexes (Table 1 and Supplementary Table S4) shows that Polβ, APE1 and XRCC1 bind BER intermediates with higher affinity than most protein partners. At the same time, Polβ and XRCC1 display similar binding affinities for each other and for the 1-nt-gapped DNA. The EC_{50} values determined here for XRCC1 and various DNA ligands are similar to the respective equilibrium dissociation constants (K_d) measured previously by intrinsic fluorescence studies of XRCC1 (37). In contrast, the apparent K_d of the Polβ and APE1 complexes with synthetic AP site-containing, gapped and nicked DNAs as determined by gel-retardation were significantly lower (in the range of 0.05 to 4 nM) (27). Such a discrepancy may result from the different techniques used. In our experiments, the lower limit of K_d was determined by the concentration of labeled protein; the lowest concentration used here to obtain reproducible data was 20 nM. Thus, the fluorescence-based approaches used in this study and by others (16,37) enable to reliably quantify high nanomolar and low micromolar affinity interactions.

Table S4. Parameters of protein-DNA interactions determined by fluorescence titration

Labeled protein[a]	DNA[b]	EC_{50}[c], nM	F_∞/F_0[c]	n[c]
TAF-Polβ	ds-DNA	160 ± 25	1.4 ± 0.1	1.4 ± 0.1
TAF-Polβ	gap-DNA	30 ± 3	3.2 ± 0.2	0.94 ± 0.02
TAF-Polβ	pUC19	2400 ± 290	5.9 ± 0.3	0.36 ± 0.05
TAF-Polβ	nick-pUC19	30 ± 4	2.2 ± 0.1	0.31 ± 0.02
FAM-Polβ	gap-DNA	23 ± 2	1.5 ± 0.1	1.3 ± 0.1
FAM-Polβ	AP-DNA	20 ± 2	1.4 ± 0.1	1.2 ± 0.1
TAF-APE1	ds-DNA	100 ± 10	2.4 ± 0.2	0.80 ± 0.1
TAF-APE1	gap-DNA	25 ± 3	2.3 ± 0.1	1.15 ± 0.05
FAM-APE1	gap-DNA	n.d.[d]	1.2 ± 0.1	n.d.[d]
FAM-APE1	AP-DNA	n.d.[d]	1.2 ± 0.1	n.d.[d]
TAF-XRCC1	ds-DNA	110 ± 12	2.1 ± 0.2	0.80 ± 0.1
TAF-XRCC1	gap-DNA	25 ± 4	2.0 ± 0.1	0.90 ± 0.05
TAF-XRCC1	pUC19	1700 ± 170	5.6 ± 0.3	0.63 ± 0.06
TAF-XRCC1	nick-pUC19	10 ± 1	1.7 ± 0.1	0.60 ± 0.05
FAM-PARP1	nick-DNA	n.d.[d]	1.1 ± 0.1	n.d.[d]
FAM-PARP1	gap-DNA	n.d.[d]	1.2 ± 0.1	n.d.[d]

[a]Titration experiments were performed at 20 nM concentration of TAF(FAM)-labeled proteins.
[b]DNA structures are shown in Supplementary Figure S1; preparation of DNA is described in the Materials and Methods section (Supplementary Data).
[c]Parameters derived from the titration curves by fitting to the four-parameter equation, where EC_{50} is the half-maximal effective concentration of DNA, at which $F - F_0 = (F_\infty - F_0)/2$; F_∞/F_0 is the extent of change in the fluorescence intensity at saturation; n is the Hill coefficient. Values are the mean (± s.d.) of three independent experiments.
[d]Parameters were not determined owing to low change in the fluorescence intensity.

Influence of BER intermediates on the protein-protein interactions

Table S5. Binding parameters of protein-protein interactions in the presence of BER intermediates

FAM-labeled protein[a]	DNA[a]	Protein partner	EC_{50}[b], nM
FAM-APE1	AP-DNA	Polβ	90 ± 7
FAM-APE1	incised AP-DNA	Polβ	170 ± 13
FAM-APE1	gap-DNA	Polβ	110 ± 8
FAM-APE1	nick-DNA	Polβ	150 ± 11
FAM-APE1	AP-DNA	XRCC1	78 ± 6
FAM-APE1	incised AP-DNA	XRCC1	83 ± 7
FAM-APE1	gap-DNA	XRCC1	51 ± 4
FAM-APE1	AP-DNA	PARP1	76 ± 7
FAM-APE1	incised AP-DNA	PARP1	68 ± 6
FAM-APE1	gap-DNA	PARP1	54 ± 5
FAM-APE1	nick-DNA	PARP1	90 ± 8
FAM-Polβ	incised AP-DNA	XRCC1	36 ± 6[c]
FAM-Polβ	gap-DNA	XRCC1	32 ± 5[c]
FAM-Polβ	nick-DNA	XRCC1	33 ± 5[c]
FAM-Polβ	incised AP-DNA	PARP1	110 ± 10
FAM-Polβ	gap-DNA	PARP1	95 ± 8
FAM-Polβ	nick-DNA	PARP1	100 ± 8
FAM-PARP1	gap-DNA	Polβ	110 ± 10
FAM-PARP1	nick-DNA	Polβ	120 ± 11
FAM-Polβ	incised AP-DNA	TDP1	300 ± 25
FAM-Polβ	gap-DNA	TDP1	250 ± 20
FAM-Polβ	nick-DNA	TDP1	280 ± 20
FAM-PARP1	gap-DNA	XRCC1	95 ± 8
FAM-PARP1	nick-DNA	XRCC1	98 ± 7

[a]Titration experiments were performed at constant concentrations of the FAM-labeled protein (40 nM) and DNA (160 nM).
[b]Parameters derived from the titration curves by fitting to the four-parameter equation, where EC_{50} is the half-maximal effective concentration of the protein partner, at which $F – F_0 = (F_\infty - F_0)/2$. Values are the mean (± s.d.) of three independent experiments.
[c]EC_{50} value for the TMR-labeled protein partner.

Analysis of protein-protein complexes by light scattering

Table S6. Analysis of protein-protein interactions by SEC-MALLS

Protein(s)[a]	Pred. Mw[b], kDa	MALLS Mw[c], kDa	Peak limits, min
Polβ	38.3	40.6	9.6 – 9.8
APE1	35.6	34.1	9.6 – 9.9
XRCC1	69.5	82.8	7.8 – 8.1
Polβ + APE1	73.9	35.3	9.6 – 9.9
XRCC1 + APE1	105.1	82.3<br>33.4	7.8 – 8.1<br>9.6 – 9.9
XRCC1 + Polβ	107.8	117.7	7.7 – 8.0
XRCC1 + Polβ + APE1	143.4	119.0<br>32.5	7.6 – 7.9<br>9.6 – 9.9

[a]Individual proteins and their equimolar mixtures were examined by SEC-MALLS. The concentration of APE1 and Polβ analyzed separately and as a mixture with each other was 120 and 60 μM respectively; the concentration of XRCC1 and its binding partners was 60 μM.
[b]Predicted (Pred.) Mw of the protomer was calculated from the protein sequence (as retrieved from the ExPASy molecular biology WWW server of SIB: http://www.expasy.org/tools/), assuming equimolar stoichiometry of hetero-oligomers.
[c]Experimentally determined weight-average Mw of species in the eluting peaks.

Table S7. Analysis of proteins and protein-protein complexes by DLS

Protein(s)[a]	Predicted R_H[b], nm		DLS R_H[c], nm	
	Protomer	Dimer	Size distribution by intensity	Size distribution by volume
Polβ	2.83	3.80	4.0 ± 0.2	3.4 ± 0.2
APE1	2.74	3.68	3.7 ± 0.2	3.1 ± 0.2
Polβ + APE1	3.74		3.8 ± 0.2	3.2 ± 0.2
XRCC1	3.65	4.90	8.5 ± 0.5	5.8 ± 0.3
XRCC1 + APE1	4.35	5.85	7.3 ± 0.4	4.5 ± 0.2
XRCC1 + Polβ	4.40	5.92	8.8 ± 0.3	6.0 ± 0.2
XRCC1 + Polβ + APE1	4.97		7.8 ± 0.4	5.4 ± 0.3

[a]Solutions of individual proteins and their equimolar mixtures at concentration of 6 μM were analyzed by DLS.
[b]Predicted hydrodynamic radius (R_H) of the protomer and of the dimer (two protomers) was calculated from the molecular weight of the constituent protein(s), assuming the globular shape of proteins and complexes.
[c]Experimentally determined average R_H of species in single peaks of the intensity and volume (mass) size distribution. Values are the mean (± s.d.) of three independent experiments.

SUPPLEMENTARY REFERENCES

1. Kubota,Y., Nash,R.A., Klungland,A., Schär,P., Barnes,D.E. and Lindahl,T. (1996) Reconstitution of DNA base excision-repair with purified human proteins: interaction between DNA polymerase β and the XRCC1 protein. *EMBO J.*, **15,** 6662–6670.
2. Marintchev,A., Robertson,A., Dimitriadis,E.K., Prasad,R., Wilson,S.H. and Mullen,G.P. (2000) Domain specific interaction in the XRCC1-DNA polymerase β complex. *Nucleic Acids Res.*, **28**, 2049–2059.
3. Marintchev,A., Gryk,M.R. and Mullen,G.P. (2003) Site-directed mutagenesis analysis of the structural interaction of the single-strand-break repair protein, X-ray cross-complementing group 1, with DNA polymerase β. *Nucleic Acids Res.*, **31**, 580–588.
4. Marintchev,A., Mullen,M.A., Maciejewski,M.W., Pan,B., Gryk,M.R. and Mullen,G.P. (1999) Solution structure of the single-strand break repair protein XRCC1 N-terminal domain. *Nat. Struct. Biol.*, **6**, 884–893.
5. Fan,J., Otterlei,M., Wong,H.K., Tomkinson,A.E. and Wilson III,D.M. (2004) XRCC1 co-localizes and physically interacts with PCNA. *Nucleic Acids Res.*, **32**, 2193–2201.
6. Akbari,M., Solvang-Garten,K., Hanssen-Bauer,A., Lieske,N.V., Pettersen,H.S., Pettersen,G.K., Wilson III,D.M., Krokan,H.E. and Otterlei,M. (2010) Direct interaction between XRCC1 and UNG2 facilitates rapid repair of uracil in DNA by XRCC1 complexes. *DNA Repair (Amst.)*, **9,** 785–795.
7. Campalans,A., Marsin,S., Nakabeppu,Y., O'connor,T.R., Boiteux,S. and Radicella,J.P. (2005) XRCC1 interactions with multiple DNA glycosylases: a model for its recruitment to base excision repair. *DNA Repair (Amst.)*, **4**, 826–835.
8. Wiederhold,L., Leppard,J.B., Kedar,P., Karimi-Busheri,F., Rasouli-Nia,A., Weinfeld,M., Tomkinson,A.E., Izumi,T., Prasad,R., Wilson,S.H., Mitra,S. and Hazra,T.K. (2004) AP endonuclease-independent DNA base excision repair in human cells. *Mol. Cell*, **15**, 209–220.
9. Das,A., Wiederhold,L., Leppard,J.B., Kedar,P., Prasad,R., Wang,H., Boldogh,I., Karimi-Busheri,F., Weinfeld,M., Tomkinson,A.E., Wilson,S.H., Mitra,S. and Hazra,T.K. (2006) NEIL2-initiated, APE-independent repair of oxidized bases in DNA: Evidence for a repair complex in human cells. *DNA Repair (Amst.)*, **5**, 1439–1448.
10. Marsin,S., Vidal,A.E., Sossou,M., Ménissier-de Murcia,J., Le Page,F., Boiteux,S., de Murcia,G. and Radicella,J.P. (2003) Role of XRCC1 in the coordination and stimulation of oxidative DNA damage repair initiated by the DNA glycosylase hOGG1. *J. Biol. Chem.*, **278**, 44068–44074.
11. Hanssen-Bauer,A., Solvang-Garten,K., Gilljam,K.M., Torseth,K., Wilson III,D.M., Akbari,M. and Otterlei,M. (2012) The region of XRCC1 which harbours the three most common nonsynonymous polymorphic variants, is essential for the scaffolding function of XRCC1. *DNA Repair (Amst.)*, **11**, 357–366.
12. Masson,M., Niedergang,C., Schreiber,V., Muller,S., Menissier-de Murcia,J. and de Murcia,G. (1998) XRCC1 is specifically associated with poly(ADP-ribose) polymerase and negatively regulates its activity following DNA damage. *Mol. Cell Biol.*, **18**, 3563–3571.

13. Beernink,P.T., Hwang,M., Ramirez,M., Murphy,M.B., Doyle,S.A. and Thelen,M.P. (2005) Specificity of protein interactions mediated by BRCT domains of the XRCC1 DNA repair protein. *J. Biol. Chem.*, **280**, 30206–30213.
14. Schreiber,V., Amé,J.C., Dollé,P., Schultz,I., Rinaldi,B., Fraulob,V., Ménissier-de Murcia,J. and de Murcia,G. (2002) Poly(ADP-ribose) polymerase-2 (PARP-2) is required for efficient base excision DNA repair in association with PARP-1 and XRCC1. *J. Biol. Chem.*, **277,** 23028–23036.
15. Loizou,J.I., El-Khamisy,S.F., Zlatanou,A., Moore,D.J., Chan,D.W., Qin,J., Sarno,S., Meggio,F., Pinna,L.A. and Caldecott,K.W. (2004) The protein kinase CK2 facilitates repair of chromosomal DNA single-strand breaks. *Cell*, **117**, 17–28.
16. Lu,M., Mani,R.S., Karimi-Busheri,F., Fanta,M., Wang,H., Litchfeld,D.W. and Weinfeld,M. (2010) Independent mechanisms of stimulation of polynucleotide kinase/phosphatase by phosphorylated and non-phosphorylated XRCC1. *Nucleic Acids Res.*, **38**, 510–521.
17. Nash,R.A., Caldecott,K.W., Barnes,D.E. and Lindahl,T. (1997) XRCC1 protein interacts with one of two distinct forms of DNA ligase III. *Biochemistry*, **36,** 5207–5211.
18. Cuneo,M.J., Gabel,S.A., Krahn,J.M., Ricker,M.A. and London,R.E. (2011) The structural basis for partitioning of the XRCC1/DNA ligase III-α BRCT-mediated dimer complexes. *Nucleic Acids Res.*, **39**, 7816–7827.
19. Plo,I., Liao,Z.Y., Barceló,J.M., Kohlhagen,G., Caldecott,K.W., Weinfeld,M. and Pommier,Y. (2003) Association of XRCC1 and tyrosyl DNA phosphodiesterase (Tdp1) for the repair of topoisomerase I-mediated DNA lesions. *DNA Repair (Amst.)*, **2**, 1087–1100.
20. Luo,H., Chan,D.W., Yang,T., Rodriguez,M., Chen,B.P., Leng,M., Mu,J.J., Chen,D., Songyang,Z., Wang,Y. and Qin,J. (2004) A new XRCC1-containing complex and its role in cellular survival of methyl methanesulfonate treatment. *Mol. Cell Biol.*, **24**, 8356–8365.
21. Dantzer,F., de La Rubia,G., Ménissier-De Murcia,J., Hostomsky,Z., de Murcia,G. and Schreiber,V. (2000) Base excision repair is impaired in mammalian cells lacking poly(ADP-ribose) polymerase-1. *Biochemistry*, **39**, 7559–7569.
22. Leppard,J.B., Dong,Z., Mackey,Z.B. and Tomkinson,A.E. (2003) Physical and functional interaction between DNA ligase IIIα and poly(ADP-ribose) polymerase 1 in DNA single-strand break repair. *Mol. Cell Biol.*, **23**, 5919–5927.
23. Das,B.B., Huang,S.Y., Murai,J., Rehman,I., Amé,J.C., Sengupta,S., Das,S.K., Majumdar,P., Zhang,H., Biard,D., Majumder,H.K., Schreiber,V. and Pommier,Y. (2014) PARP1-TDP1 coupling for the repair of topoisomerase I-induced DNA damage. *Nucleic Acids Res.*, **42**, 4435–4449.
24. Dimitriadis,E.K., Prasad,R., Vaske,M.K., Chen,L., Tomkinson,A.E., Lewis,M.S. and Wilson,S.H. (1998) Thermodynamics of human DNA ligase I trimerization and association with DNA polymerase β. *J. Biol. Chem.*, **273**, 20540–20550.
25. Whitehouse,C.J., Taylor,R.M., Thistlethwaite,A., Zhang,H., Karimi-Busheri,F., Lasko,D.D., Weinfeld,M. and Caldecott,K.W. (2001) XRCC1 stimulates human polynucleotide kinase activity at damaged DNA termini and accelerates DNA single-strand break repair. *Cell*, **104**, 107–117.

26. Bennett,R.A., Wilson III,D.M., Wong,D. and Demple,B. (1997) Interaction of human apurinic endonuclease and DNA polymerase β in the base excision repair pathway. *Proc. Natl Acad. Sci. USA*, **94**, 7166–7169.
27. Liu,Y., Prasad,R., Beard,W.A., Kedar,P.S., Hou,E.W., Shock,D.D. and Wilson,S.H. (2007) Coordination of steps in single-nucleotide base excision repair mediated by apurinic/apyrimidinic endonuclease 1 and DNA polymerase β. *J. Biol. Chem.*, **282**, 13532–13541.
28. Chiang,S.C., Carroll,J. and El-Khamisy,S.F. (2010) TDP1 serine 81 promotes interaction with DNA ligase IIIα and facilitates cell survival following DNA damage. *Cell Cycle*, **9**, 588–595.
29. El-Khamisy,S.F., Masutani,M., Suzuki,H. and Caldecott,K.W. (2003) A requirement for PARP-1 for the assembly or stability of XRCC1 nuclear foci at sites of oxidative DNA damage. *Nucleic Acids Res.*, **31**, 5526–5533.
30. Langelier,M.F., Planck,J.L., Roy,S. and Pascal,J.M. (2012) Structural basis for DNA damage-dependent poly(ADP-ribosyl)ation by human PARP-1. *Science*, **336**, 728–732.
31. Sawaya,M.R., Pelletier,H., Kumar,A., Wilson,S.H. and Kraut,J. (1994) Crystal structure of rat DNA polymerase β: evidence for a common polymerase mechanism. *Science*, **264**, 1930–1935.
32. Cotner-Gohara,E., Kim,I.K., Tomkinson,A.E. and Ellenberger,T. (2008) Two DNA-binding and nick recognition modules in human DNA ligase III. *J. Biol. Chem.*, **283**, 10764–10772.
33. Bernstein,N.K., Williams,R.S., Rakovszky,M.L., Cui,D., Green,R., Karimi-Busheri,F., Mani,R.S., Galicia,S., Koch,C.A., Cass,C.E., Durocher,D., Weinfeld,M. and Glover,J.N. (2005) The molecular architecture of the mammalian DNA repair enzyme, polynucleotide kinase. *Mol. Cell*, **17**, 657–670.
34. Kutuzov,M.M., Khodyreva,S.N., Amé,J.C., Ilina,E.S., Sukhanova,M.V., Schreiber,V. and Lavrik,O.I. (2013) Interaction of PARP-2 with DNA structures mimicking DNA repair intermediates and consequences on activity of base excision repair proteins. *Biochimie*, **95**, 1208–1215.
35. Arndt,J.W., Gong,W., Zhong,X., Showalter,A.K., Liu,J., Dunlap,C.A., Lin,Z., Paxson,C., Tsai,M.D. and Chan,M.K. (2001) Insight into the catalytic mechanism of DNA polymerase β: structures of intermediate complexes. *Biochemistry*, **40**, 5368–5375.
36. Gorman,M.A., Morera,S., Rothwell,D.G., de La Fortelle,E., Mol,C.D., Tainer,J.A., Hickson,I.D. and Freemont,P.S. (1997) The crystal structure of the human DNA repair endonuclease HAP1 suggests the recognition of extra-helical deoxyribose at DNA abasic sites. *EMBO J.*, **16**, 6548–6558.
37. Mani,R.S., Karimi-Busheri,F., Fanta,M., Caldecott,K.W., Cass,C.E. and Weinfeld,M. (2004) Biophysical characterization of human XRCC1 and its binding to damaged and undamaged DNA. *Biochemistry*, **43**, 16505–16514.
38. Cuneo,M.J. and London,R.E. (2010) Oxidation state of the XRCC1 N-terminal domain regulates DNA polymerase β binding affinity. *Proc. Natl Acad. Sci. USA*, **107**, 6805–6810.

SUPPLEMENTARY FIGURES

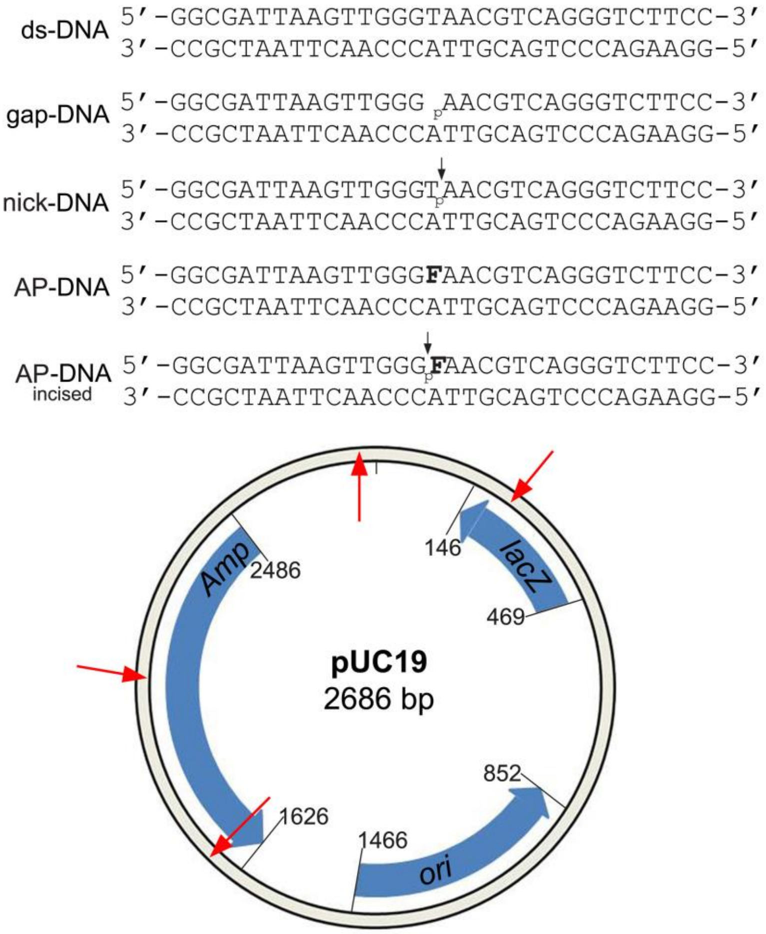

Figure S1. Structures of DNA ligands used in fluorescence titration experiments. Sites of the pUC19 cleavage by nickase N.Bst9I are shown by arrows.

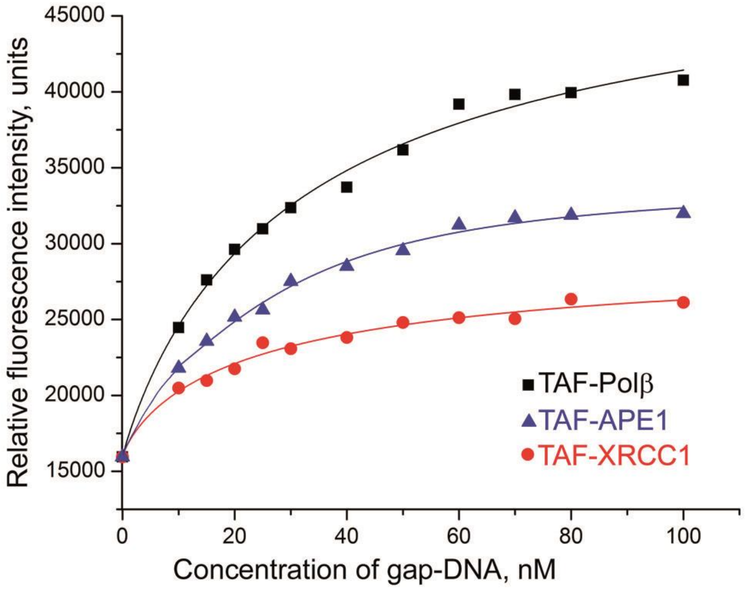

Figure S2. Fluorescence titration of TAF-labeled Polβ, APE1 and XRCC1 with 1-nt-gapped DNA. The TAF-labeled protein (20 nM) was excited at 485 nm in the absence or presence of increasing concentrations of the BER intermediate and the relative fluorescence intensities were monitored at 520 nm. Curves show the best fits of the four-parameter equation; R^2 values meet or exceed 0.97.

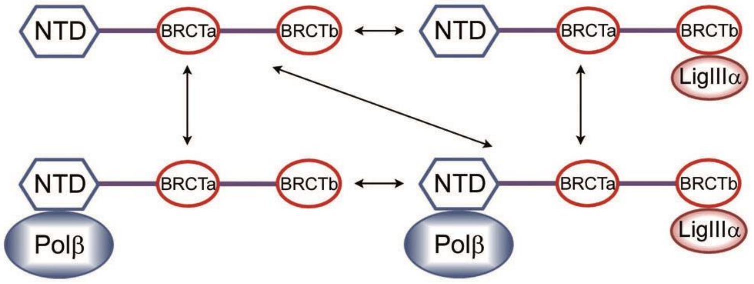

Figure S3. Schematic representation of XRCC1 hetero-oligomerization with Polβ and LigIIIα. The XRCC1 domains (NTD, BRCTa and BRCTb) and domains of Polβ (catalytic domain) and LigIIIα (BRCT) interacting with XRCC1 are depicted proportionally to their dimensions in the X-ray structures (18,38).
